# Revisiting Ancient Polyploidy in Leptosporangiate Ferns

**DOI:** 10.1111/nph.18607

**Published:** 2022-11-08

**Authors:** Hengchi Chen, Yuhan Fang, Arthur Zwaenepoel, Sanwen Huang, Yves Van de Peer, Zhen Li

**Affiliations:** 1 Department of Plant Biotechnology and Bioinformatics, Ghent University, 9052 Ghent, Belgium; 2 VIB Center for Plant Systems Biology, VIB, 9052 Ghent, Belgium; 3 Laboratory for Lingnan Modern Agriculture, Genome Analysis Laboratory of the Ministry of Agriculture and Rural Affairs, Agricultural Genomics Institute at Shenzhen, Chinese Academy of Agricultural Sciences, Shenzhen, Guangdong 518124, China; 4 Centre for Microbial Ecology and Genomics, Department of Biochemistry, Genetics and Microbiology, University of Pretoria, Pretoria 0028, South Africa; 5 College of Horticulture, Academy for Advanced Interdisciplinary Studies, Nanjing Agricultural University, Nanjing, Jiangsu 210095, China

**Keywords:** ferns, gene tree – species tree reconciliation, *K*_s_-age distribution, phylogenomics, polyploidy, WGD

## Abstract

- Ferns, and particularly homosporous ferns, have long been assumed to have experienced recurrent whole-genome duplication (WGD) events because of their substantially large genome sizes, surprisingly high chromosome numbers, and high degrees of polyploidy among many extant members. As the number of sequenced fern genomes is limited, recent studies have employed transcriptome data to find evidence for WGDs in ferns. However, they have reached conflicting results concerning the occurrence of ancient polyploidy, for instance, in the lineage of leptosporangiate ferns.
- Because identifying WGDs in a phylogenetic context is the foremost step in studying the contribution of ancient polyploidy to evolution, we here revisited earlier identified WGDs in leptosporangiate ferns, mainly the core leptosporangiate ferns, by building *K_s_*-age distributions and applying substitution rate corrections, and by conducting statistical gene tree – species tree reconciliation analyses.
- Our integrative analyses confidently identified four ancient WGDs in the sampled core leptosporangiate ferns but also identified false positives and false negatives for WGDs that recent studies have reported earlier.
- In conclusion, we underscore the significance of substitution rate corrections and uncertainties in gene tree – species tree reconciliations in calling WGD events and advance an exemplar workflow to overcome such often-overlooked issues.

## Introduction

Tracheophytes, or vascular plants, have shaped the diversity of the terrestrial ecosystem on Earth since their first appearance about 431 to 451 million years ago (Morris *et al*., 2018). Tracheophytes are composed of three major groups, spermatophytes (seed plants), Lycopodiopsida (lycophytes), and Polypodiopsida (ferns) (PPG I, 2016). Seed plants form a monophyletic group, including gymnosperms and angiosperms. Within the past two decades, strong evidence has accumulated for recurrent paleo-polyploidizations, or ancient whole-genome duplications (WGDs) in seed plants (Cui *et al*., 2006; Vekemans *et al*., 2012; Vanneste *et al*., 2014; Ming *et al*., 2015; Van de Peer *et al*., 2017; Stull *et al*., 2021) and their importance for the evolution of innovative traits and in facilitating the diversification of seed plant species are undisputed (Soltis & Soltis, 2016; Van de Peer *et al*., 2017; Landis *et al*., 2018; Fox *et al*., 2020; Van de Peer *et al*., 2021). Different from the lineage of seed plants, strong evidence for WGDs in lycophytes and ferns was lacking, although cytological evidence suggested that polyploidization may not be uncommon in ferns (Wood *et al*., 2009; Clark *et al*., 2016; Wang *et al*., 2022).

Ferns are the largest group of non-seed vascular plants and make up more than 90% of the extant diversity (PPG I, 2016). Compared to seed plants, very few have had their genome sequenced so far (Szövényi *et al*., 2021), because they, especially homosporous ferns, tend to have large genome sizes and large to huge chromosome numbers. For example, the modern fern or C-fern (*Ceratopteris richardii*) possesses 2n = 78 chromosomes with a genome of 11.25 Gbp (Marchant *et al*., 2019). More strikingly, *Ophioglossum reticulatum* is a fern species with the highest chromosome number known amongst eukaryotes, with 2n = 1,440 chromosomes (Khandelwal, 1990). The huge diversity as well as the high numbers of chromosomes of ferns are compelling mysteries that have fascinated evolutionary biologists for decades (Haufler & Soltis, 1986; Barker, 2009; Clark *et al*., 2016; Wang *et al*., 2022). Given that polyploidizations can increase both genome sizes and chromosome numbers directly, multiple rounds of polyploidizations, along with potential changes in chromosome compositions and/or processes of genome downsizing, have been hypothesized to explain the evolution of chromosomes and genomes (Clark *et al*., 2016; Wang *et al*., 2022) and further the species diversity in ferns (Fujiwara *et al*., 2021).

It is not until recently that genomic and transcriptomic data have begun to shed light on ancient polyploidies in ferns (Barker & Wolf, 2010; Szövényi *et al*., 2021). Analyses of the first two genomes of heterosporous ferns, *Azolla filiculoides* and *Salvinia cucullata*, have identified two WGDs, with one specific to the genus *Azolla* and the other shared by all core Leptosporangiates (Li *et al*., 2018). Furthermore, the flying spider-monkey tree fern (*Cyathea spinulosa*) genome has unveiled WGDs in Cyatheales (Huang *et al*., 2022), while the partial genome of the C-fern has provided some evidence for a WGD also in its lineage (Marchant *et al*., 2019). Also, analyses of *Equisetum* transcriptomes showed ancient polyploidy in the lineage (Vanneste *et al*., 2015; Clark *et al*., 2019). In addition, two recent studies have added valuable supplements of transcriptome data to the scarce genomic data of ferns and suggested several ancient WGDs during the evolutionary history of ferns (1KP initiative, 2019; Huang *et al*., 2020). However, conflicting results regarding the identified WGD events in ferns have been proposed in the previous studies.

**Fig. 1:**
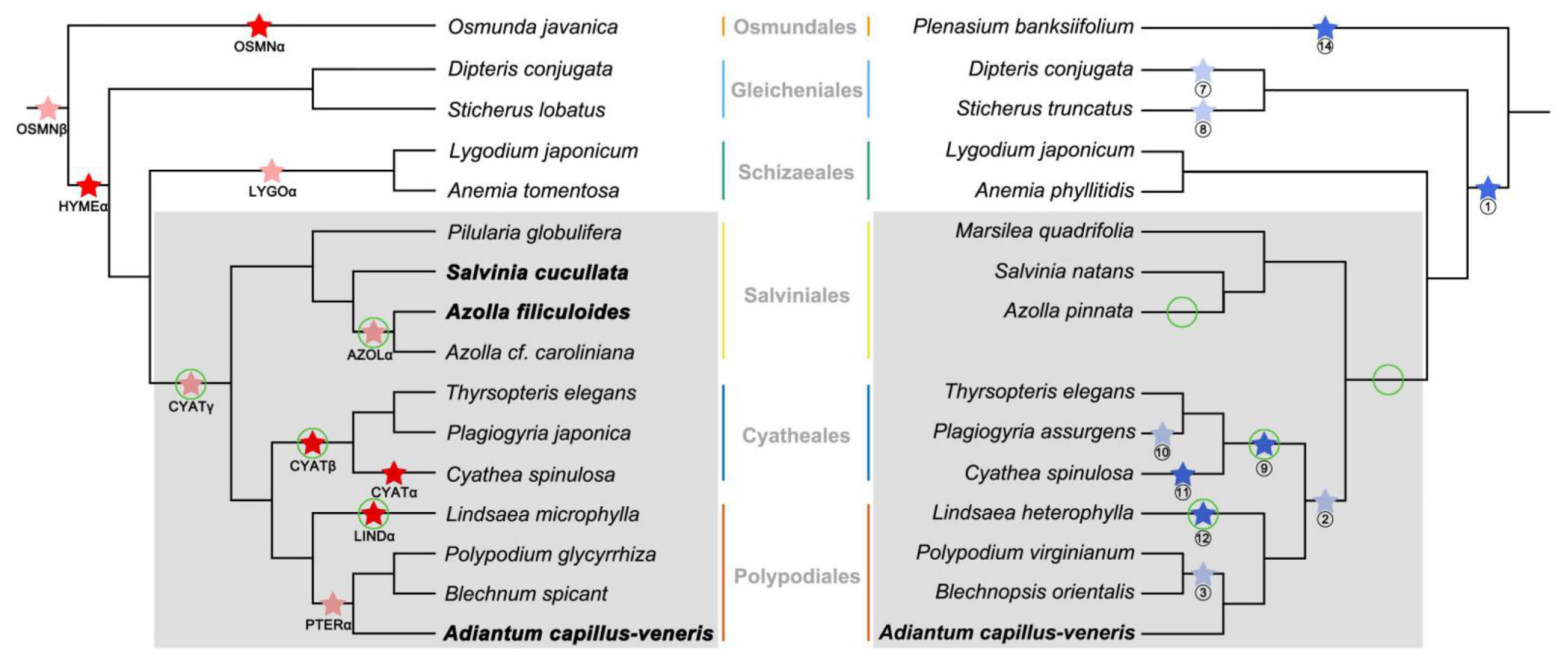
Identified WGD events in leptosporangiates, as reported by the 1KP initiative (2019) (left) and by Huang et al. (2020) (right). Ten of 14 WGDs in leptosporangiates from the 1KP initiative (2019) are denoted as (light) red stars. Four WGDs are not included because they were placed in lineages not studied by Huang et al. (2020). Ten out of ten WGD events from Huang et al. (2020) are denoted as (light) blue stars. WGD events found in the same lineages by both studies are in solid red and solid blue. The grey background highlights the core leptosporangiates in the two phylogenetic trees. The names for species with fully sequenced genomes are in bold. The green circles denote the WGDs in core leptosporangiates identified in this study.

Here, we revisited the occurrence of WGDs in leptosporangiate ferns, or more specifically in the lineage of core leptosporangiates, for leptosporangiate ferns form most of the species in extant ferns (Pryer *et al*., 2004). Leptosporangiates are subdivided into seven orders, namely Osmundales (*c*. 18 species), Hymenophyllales (*c*. 434 species), Gleicheniales (*c*. 172 species), Schizaeales (*c*. 190 species), Salviniales (*c*. 82 species), Cyatheales (*c*. 713 species), and Polypodiales (*c*. 8,714 species), the last three of which include most species and constitute the lineage of core leptosporangiates (Smith *et al*., 2006; PPG I, 2016). To focus on those ferns that have been investigated in previous studies with a well-resolved phylogeny (Shen *et al*., 2018), we selected species in all three orders of the core leptosporangiates and used representatives of another three leptosporangiate orders as outgroups. This way, we could revisit ten out of 14 WGDs reported by the 1KP initiative (2019) and all ten WGDs reported by Huang et al. (2020) in leptosporangiates. For the ten WGD events retrieved from each study, only five are congruent and have been placed in the same phylogenetic position (Fig. 1).

The conflicting results concerning the identified WGDs in previous studies could result from several pitfalls that are often overlooked in the two commonly used approaches to find evidence for ancient WGDs, namely *K*_s_-age distributions of paralogs and gene tree – species tree reconciliation approaches. Although these two approaches have great power and have been widely applied to detect WGDs based on genomic and transcriptome data (Jiao *et al*., 2011; Vanneste *et al*., 2013; Li *et al*., 2015; McKain *et al*., 2016; Zhang *et al*., 2017; Ren *et al*., 2018), they must be used with caution (Tiley *et al*., 2018; Zwaenepoel *et al*., 2019; Zwaenepoel & Van de Peer, 2019). WGDs in so-called *K*_s_-age distributions, where the number of duplicates is plotted against their age as inferred from the expected number of synonymous substitutions per synonymous site (*K*_s_), can be identified as peaks in the distribution, which suggest that many genes have been duplicated at the same time (Vanneste *et al*., 2013). Such *K*_s_ peaks are often compared with speciation events characterized by *K*_s_ distributions of orthologs between species to infer the relative or absolute timing of the WGDs. However, such comparisons admit meaningful interpretation only if substitution rates of the species under consideration are similar, while substitution rates naturally vary across lineages. It has been gradually acknowledged that different substitution rates can affect the placement of WGDs (Barker *et al*., 2008; Chen *et al*., 2020; Sensalari *et al*., 2021). For instance, if two species sharing one WGD diverged and have evolved at different substitution rates afterward, given no correction for the difference in substitution rates, the WGD *K*_s_ peak identified in the species with a lower substitution rate may be incorrectly interpreted as a younger and lineage-specific WGD. In contrast, species with a higher substitution rate may still support a shared WGD. This could eventually lead to erroneous conclusions, especially when no genome is available to determine the inference of WGDs via collinear analysis.

A second approach to identify and date WGDs is to use gene tree – species tree reconciliation, where events underlying the evolutionary history of a gene, like gene duplication and loss, hybridization, introgression, horizontal gene transfer, and incomplete lineage sorting, are identified by mapping gene trees onto species trees. When many duplicated genes are reconciled on one specific branch of the species tree, this can be considered evidence for a WGD. Although the 1KP initiative (2019) and Huang et al. (2020) have implemented the reconciliation approaches differently, both have employed the least common ancestor (LCA) reconciliation to determine duplication events on a species phylogeny based on gene trees inferred by maximum likelihood (ML) inference. In LCA reconciliation, a duplication event involving genes from some species is placed on a species phylogeny at the node associated with the most recent common ancestor of these species (Zmasek & Eddy, 2001). Even if gene trees have been filtered based on their quality before reconciliations (based on bootstrap support values, for instance), the LCA reconciliation is still error-prone in placing gene duplication and loss events, and its accuracy depends on the correctness of inferred gene tree topologies (Hahn, 2007). Nevertheless, the true gene tree topology for a gene family is often one among many statistically equivalent gene trees (Wu *et al*., 2013), so only considering the one ‘best’ ML tree for each gene family may cause systematic bias when using LCA reconciliation to identify WGDs (Hahn, 2007; Zwaenepoel & Van de Peer 2019). In addition, a WGD and its phylogenetic position are often determined when the number of duplication events on a branch exceeds a certain cut-off, which is usually set somewhat arbitrarily without acknowledging the varying contribution of small-scale duplications (SSDs) along different branches of the species tree (Li *et al*., 2015; McKain *et al*., 2016; Ren *et al*., 2018), which may result in false positive WGD identification towards the tips of a species phylogeny (Zwaenepoel & Van de Peer, 2019).

To revisit ancient polyploidy in leptosporangiate ferns, we retrieved relevant transcriptome data from the 1KP initiative (2019), for its relatively high quality and reasonably high gene numbers (Fig. S1). Also, we added two publicly available genomes of heterosporous ferns and a newly sequenced homosporous fern, *Adiantum capillus-veneris* L. (Fang *et al*., 2022). We did not include the C-fern genome because of its partial and fragmented nature (Marchant *et al*., 2019). By considering differences in substitution rates and performing statistical gene tree – species tree reconciliations under a model integrating small-scale gene duplication and loss (DL) and WGDs, we confidently identified four WGDs in core leptosporangiate ferns (Fig. 1), fewer than the six WGDs as found by 1KP initiative (2019) and Huang et al. (2020), while some WGDs have also been predicted at different phylogenetic positions, suggesting that some WGDs identified by the two previous studies are likely false positives. Our study again highlights the importance of fully recognizing the caveats and limitations of commonly used approaches in calling WGD events.

## Materials and Methods

### Transcriptomes and Genomes of Leptosporangiates

We selected 16 and 15 species and their corresponding assembled transcriptomes from the 1KP initiative (2019) and Huang et al. (2020), respectively. Except for the order Hymenophyllales due to its uncertain phylogenetic position (PPG I, 2016;1KP initiative, 2019), the remaining six orders in leptosporangiate ferns were sampled (Fig. 1; Table S1). We removed unigenes with identical sequences by SeqKit (v0.7.1) (Shen *et al*., 2016) and filtered out coding sequences that were not divisible by three or had unknown nucleotides or premature stop codons. We retrieved the genomes of *Azolla filiculoides* and *Salvinia cucullata* from fernbase.org (Li *et al*., 2018) and used the *Adiantum capillus-veneris* genome (Fang *et al*., 2022). We then used BUSCO (v4.0.2) to assess gene space completeness in the three ferns with complete genomes and the 13 ferns with 1KP transcriptomes using embryophyta_odb10 (Simão *et al*., 2015; Kriventseva *et al*., 2019) (Fig. S2).

### Constructing *K*_s_-age distributions

*K*_s_-age distributions for all paralogous genes (paranome) in transcriptomes and genomes were constructed by wgd (v1.1.1) (Zwaenepoel & Van de Peer, 2018). To detect peaks that could be signatures of WGD events in the *K*_s_ distributions, we performed mixture modeling using the R package mclust (v5.4.7) (Scrucca *et al*., 2016). We first transformed *K*_s_ distributions into log-scale, which were further fitted to a serial of Gaussian mixture models (GMM) (Rasmussen, 1999). We increasingly fitted one to eight components per mixture model and used the Bayesian Information Criterion (BIC) to select the optimal number of components. Although BIC strongly penalizes increases in the number of parameters, the GMM is still prone to overfitting, so we further performed SiZer (Significance of Zero Crossings of the Derivative) analysis using the R package feature (v1.2.15) (https://cran.r-project.org/web/packages/feature/index.html) to distinguish *bona fide* peaks in the *K*_s_ distributions from those that represent noises (Chaudhuri & Marron, 1999) (Fig. S3, S4).

### Correcting differences in synonymous substitution rates

Orthologous *K*_s_ distributions were constructed by wgd (Zwaenepoel & Van de Peer, 2018). To circumscribe the phylogenetic placements of the identified WGDs in the *K*_s_-age distributions for paranomes, we corrected the differences in synonymous substitution rates across species using two approaches. In the first approach, we used OrthoFinder (v2.3.3) (Emms & Kelly, 2019) with default settings, except “-M msa”, to identify gene families with the 16 species in Fig. 1. We used MUSCLE (v3.8.31) (Edgar, 2004) to perform multiple sequence alignment of the proteins for the 34 identified single-copy gene families, which were further concatenated after being trimmed and back-translated by trimal (v1.4.1) (Capella-Gutiérrez *et al*., 2009). PAML (v4.9j) with the free-ratio model (Yang, 2007) was then used to estimate branch lengths in *K*_s_ unit for the species phylogeny. To map all the identified *K*_s_ peaks onto the species phylogeny in *K*_s_ units, we halved *K*_s_ values of the identified peaks in the GMM analyses (Fig. S3) and placed each peak from the tip towards the root of the phylogeny to date when WGD events have occurred in the phylogeny, with the assumption that duplicate genes on average evolved at similar substitution rates after WGD events.

In the second approach, we used ksrates (v1.0), which corrects synonymous substitution rates of other species to the rate of a focal species, i.e., the species desired to implement comparisons between the relative date of WGD and species divergence (Sensalari *et al*., 2021). To identify peaks representing WGDs, ksrates fits an exponential-lognormal mixture model to each *K*_s_-age distribution, using the BIC to evaluate one exponential component for the L-shaped SSDs (Lynch & Conery, 2003) and one to five lognormal components for potential WGD peaks. Then, ksrates compared WGD peaks and corrected orthologous *K*_s_ peaks to infer the timing of WGDs (Fig. S5). Besides using default parameters, we set a maximum of 14 sets of trios to correct each divergence with multiple outgroups and used means to form consensus divergence peaks.

### Statistical Gene Tree – Species Tree Reconciliation

To prepare data for the reconciliation analysis, we retrieved a species tree with divergence times from TimeTree (Kumar *et al*., 2017) (Fig. S6). Based on the 16,305 gene families identified above, we filtered 9,442 gene families for large family sizes or no common ancestor at the root by “orthofilter.py” (https://github.com/arzwa/Whale.jl). We then used PRANK (v150803) (Löytynoja, 2014) to perform multiple sequence alignment for each gene family and MrBayes (v.3.2.6) (Ronquist *et al*., 2012) to infer posterior probability distributions of gene trees under the LG+GAMMA model. MrBayes ran 110,000 generations and sampled at a frequency of 10 to get in total 11,000 posterior samples for each of the 6,863 gene families. Lastly, ALEobserve (Szöllõsi *et al*., 2013) constructed the conditional clade distribution containing marginal clade frequencies with a burn-in of 1,000.

Using Whale (v2.0.3) (Zwaenepoel & Van de Peer, 2019), we carried out statistical gene tree – species tree reconciliation and tested the occurrences of eight WGDs (Fig. S6) under the so-called DL+WGD model, which considers both small-scale gene duplication and loss (DL), and WGDs. Two DL+WGD models were adopted to incorporate various DL rates of SSDs across the species tree (Methods S1). In the critical branch-specific DL+WGD model, where we assumed the duplication (*λ*) and loss rates (*μ*) to be equal on each branch, a *Beta*(3,1) prior distribution was used for *η*, the parameter of the geometric prior distribution on the number of genes at the root. We used an improper flat prior for the mean branch rate *r*. The branch rates were assumed to follow a multivariate Gaussian prior with an Exponential prior with mean 0.1 for the standard deviation. For the more flexible DL+WGD model with branch-specific DL rates model, we assumed an independent bivariate normal prior with mean 0 and standard deviation 1 for the mean log-DL rate, assumed a *Uniform*(-1,1) prior for the correlation coefficient of DL rate for each individual branch, and assumed an exponential prior with mean 1 for the standard deviation of the branch rates. Lastly, because most of the analyzed species only have transcriptome data, missing genes were considered in the models by leveraging the BUSCO missing values of each species (Fig. S2). Generally, the effective sample sizes (ESSs) of all the inferred parameters were over 200, with a median over 500, indicating good approximations of the posteriors (Table S2, S3). We further checked the convergence of parameters with a subset of the 6,863 gene families by randomly selecting 1,000 gene families. We inferred their gene trees using MrBayes with 1,100,000 generations and a sampling frequency of 100. We filtered out 124 gene families (Fig. S7) and ran Whale under both models. The 95% uncertainty intervals for most of the estimates based on the subset overlapped with the ones based on the 6,863 gene families (Table S4, S5).

### Collinear Analysis of Available Fern Genomes

We used i-ADHoRe (v.3.0.01) (Proost *et al*., 2011) to delineate both intra- and intergenomic collinearity with the three available fern genomes. For the intragenomic comparisons, i-ADHoRe in wgd identified 361, 414, and 375 anchor pairs – duplicate pairs retained in the collinear regions – in the genomes of *A*. *capillus-veneris*, *A*. *filiculoides*, and *S*. *cucullata*, respectively. *K*_s_ distributions for the anchor pairs show a larger fractions of anchor pairs with *K*_s_ values ranging from 0 to 0.1 in *A. filiculoides* and *S. cucullata*, compared to those in *A. capillus-veneris* (Fig. S8). Most of the anchor pairs with small *K*_s_ values are located on short scaffolds in the *A. filiculoides* and *S. cucullata* assemblies (Fig. S9), reflecting that they are still fragmented to a certain extent. We hence removed short scaffolds with fewer than ten genes and anchor pairs with *K*_s_ values less than 0.1 for intergenomic comparisons. To infer collinear ratios among the three fern genomes, we performed all-against-all BLASTP (v.2.6.0+) (Camacho *et al*., 2009) for all proteins from the three ferns with an *E*-value of 1 *×* 10^-5^ and ‘-max_target_seqs = 100000’. Homologous pairs were filtered using the *c*-score of 0.5 (Putnam *et al*., 2007) and were fed into i-ADHoRe to analyze intergenomic collinear ratio (Fig. S9).

Also, we used Whale without hypothetical WGD events to perform gene tree – species tree reconciliations for gene families having anchor pairs to estimate the expected number of duplication and loss on each branch (Methods S2). We fixed the *η* parameter to 0.75 based on the average number of observed genes in a family. An exponential prior with *r* for the expected number of duplication/loss events along a branch was assumed, where a noninformative prior was set for *r*. To reserve duplicates that were likely derived from WGD events, we kept 30, 75, and 36 anchor pairs in 137 gene families, which had a common ancestor at the root of the species tree and whose *K*_s_ values fell in the ranges of [1.8,2.5], [0.6,1.2] and [1.0,1.9] according to the paranome *K*_s_-age distributions of *A*. *capillus-veneris*, *A*. *filiculoides*, and *S*. *cucullata*, respectively (Fig. S8).

## Results

### Different substitution rates among ferns in core leptospongiates

**Fig. 2:**
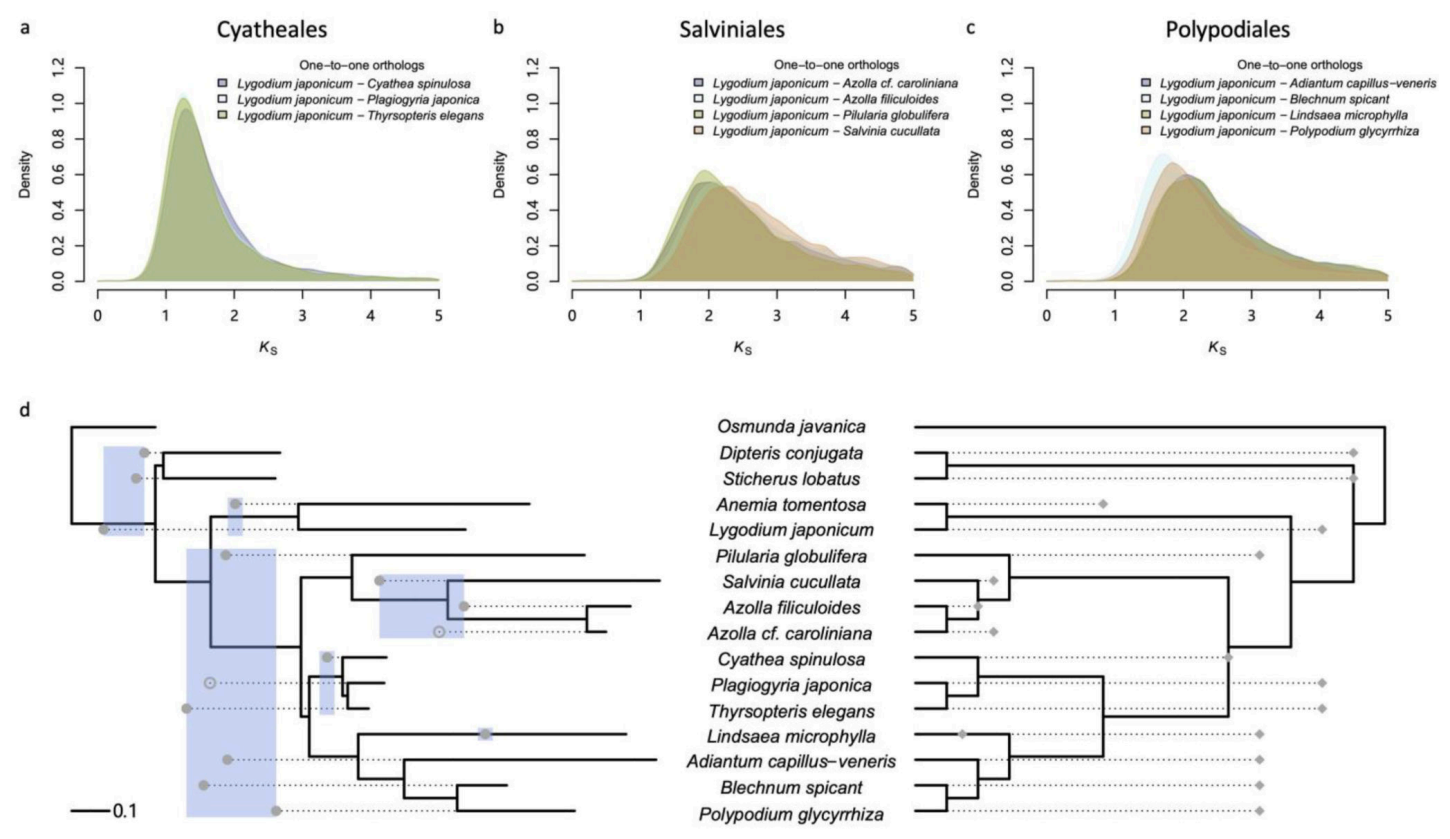
Orthologous *K*_s_ distributions and WGD events identified based on *K*_s_ distributions for the whole paranomes of different fern species. (a-c) One-to-one orthologous *K*_s_ distributions between *Lygodium japonicum* and species from Cyatheales (a), Salviniales (b), and Polypodiales (c). (d) WGD events identified based on *K*_s_ distributions for the whole paranome of species in the phylogeny. On the left, a species phylogram is shown with branch lengths in *K*_s_ units, while WGD events are depicted as dots (calculated as half the *K*_s_ peak value of each species starting from the corresponding tip). Solid dots denote significant *K*_s_ peaks in the SiZer analysis, whereas hollow dots denote *K*_s_ peaks only identified by GMM but not SiZer (see Materials and Methods and Fig. S3, S4). On the right, a species cladogram is shown, where WGD events are depicted as rhombs according to the analyses of ksrates (Sensalari et al. 2021). Note that when a WGD and a speciation event overlap in the ksrates analysis, the WGD event is placed at the speciation event in the cladogram.

To compare synonymous substitution rates among core leptosporangiate ferns, we compared one-to-one orthologous *K*_s_ distributions between *Lygodium japonicum* from Schizaeales (or *Dipteris conjugata* from Gleicheniales in Fig. S10) and species from Cyatheales, Salviniales, and Polypodiales within core leptospongiates (Fig. 2a-c). Because peaks in the orthologous *K*_s_ distributions all represent the divergence between Schizaeales and the core leptospongiates, they should have identical or at least very similar *K*_s_ peak values if the selected species all have similar substitution rates. However, the orthologous *K*_s_ peak values are smaller for species from Cyatheales than species from Salviniales and Polypodiales, suggesting that nuclear genes in Cyathealean species, like chloroplast genes (Zhong *et al*., 2014), have slower substitution rates than their counterparts from the other two orders in the core leptosporangiates. In addition, the orthologous *K*_s_ peak values for the species belonging to Salviniales and Polypodiales show more variation than the ones in Cyatheales, indicating more variable substitution rates among Salvinialean and Polypodialean species (Fig. 2a-c).

### Inferring WGDs by applying substitution rate corrections

As substitution rates affect *K*_s_ distributions, peaks in the paralogous *K*_s_ distributions may differ for species with different substitution rates, even if they have experienced the same WGD event. We hence wondered if the differences in calling WGDs within core leptosporangiates in previous studies could be due to different substitution rates. *K*_s_-age distributions for the whole paranomes of the 16 selected species, except for *Plagiogyria japonica* and *Azolla cf. caroliniana*, all show a peak verified by the SiZer analyses (Fig. S3, S4). The peaks show various *K*_s_ values and are largely in line with the paranome *K*_s_ distributions from the 1KP initiative (2019).

To correct for different synonymous substitution rates among species, we adopted two recently developed approaches. In the first approach, we inferred a species phylogeny in *K*_s_ units so that we could compare WGD peaks in one species with its divergence from other species (Chen *et al*., 2020). If we assume that both paralogs, on average, evolved at a similar rate after a WGD event, we could simply consider half the *K*_s_ values of all identified peaks in each species to position, starting from each tip, the WGDs on the phylogenetic tree (Fig. 2d). By applying this approach, we show that the *K*_s_ peak identified in *Lindsaea microphylla* supports one WGD in the lineage of *Lindsaea* (Fig. 2d), while the *K*_s_ peaks found in *Blechnum spicant*, *Polypodium glycyrrhiza*, and *Adiantum capillus-veneris* all support a more ancient WGD likely shared by the core leptospongiates (Fig. 2d), although they all have different *K*_s_ peak values (Fig. S3) for different substitution rates (Fig. 2c; Fig. S10).

In Cyatheales, the *K*_s_ peak in *Cyathea spinulosa* supports a WGD shared by Cyathales. If true, we would expect to observe clear *K*_s_ peaks in *Thyrsopteris elegans* and *Plagiogyria japonica* as well. Unexpectedly, only a *K*_s_ peak supporting a WGD before the divergence of core leptospongiates and Schizaeales has been observed in *Thyrsopteris elegans* (Fig. 2d). Similarly, a less perceptible *K*_s_ peak in *Plagiogyria japonica*, which is supported by the GMM but the SiZer analysis, also suggests an ancient WGD event. We argue that *Thyrsopteris elegans* and *Plagiogyria japonica* have even lower synonymous substitution rates than *Cyathea spinulosa* (Fig. 2a). If the *K*_s_ peak for the Cyathealean WGD is at *K*_s_≈ 0.3 in the paranome *K*_s_distribution of *Cyathea spinulosa*, the expected *K*_s_peaks in slower evolving *Thyrsopteris elegans* and *Plagiogyria japonica* must have even smaller values, which may be confounded by the background *K*_s_distribution from SSDs (Fig. S11).

In Salviniales, the *K*_s_ peak in *Azolla filiculoides* supports a WGD in the lineage leading to *Azolla*. However, the paranome *K*_s_ distribution of *Salvinia cucullata* has a peak supporting a WGD before the divergence of *Salvinia* and *Azolla*, instead of a WGD before the divergence of core leptosporangiates, as suggested earlier (Li *et al*., 2018). Also, the GMM has disentangled a peak in the paranome *K*_s_ distribution of *Azolla cf. caroliniana*, suggesting a WGD shared by *Salvinia* and *Azolla*, but the peak is not significant in the SiZer analysis (Fig. S3). Lastly, a WGD shared by core leptosporangiates is evidenced by the *K*_s_ peak found in *Piluaria globulifera*.

In the second approach, we used ksrates, which adjusts synonymous substitution rates to the rate of a focal species by relative rate tests (Sensalari *et al*., 2021). Therefore, peaks identified in the *K*_s_-age distribution of the focal species can be directly compared with speciation events represented by orthologous *K*_s_ distributions (Fig. S5). By correcting for unequal substitution rates among species, the ksrates analysis confirms our previous results, except that the ksrates analysis provides extra support from *Lindsaea microphylla* for a WGD shared by the core leptospongiates (Fig. 2d).

### Evaluating WGDs with two different DL+WGD models

The analyses of *K*_s_-age distributions as described above suggest eight branches in the species phylogeny potentially associated with WGDs, but the occurrence of WGDs on some branches is ambiguous, because *K*_s_ peaks from different species fall onto adjacent branches (Fig. 2d). For example, the *K*_s_ peaks from *Polypodium glycyrrhiza*, *Pilularia globulifera*, and *Adiantum capillus-veneris* support a WGD shared by all core leptospongiates, whereas the ones from *Thyrsopteris elegans*, *Blechnum spicant*, and *Plagiogyria japonica* support a WGD before the divergence between core leptospongiates and Schizaeales. Similarly, the *K*_s_ peak from *Azolla filiculoides* supports a WGD specific to *Azolla*. Still, the *K*_s_ peak for *Salvinia cucullata* favors a shared WGD by *Azolla* and *Salvinia*. These results could point to two independent WGDs, one before and one after the speciation event, or alternatively, to one WGD event that is, however, represented by *K*_s_ peaks with different values for different species.

**Fig. 3:**
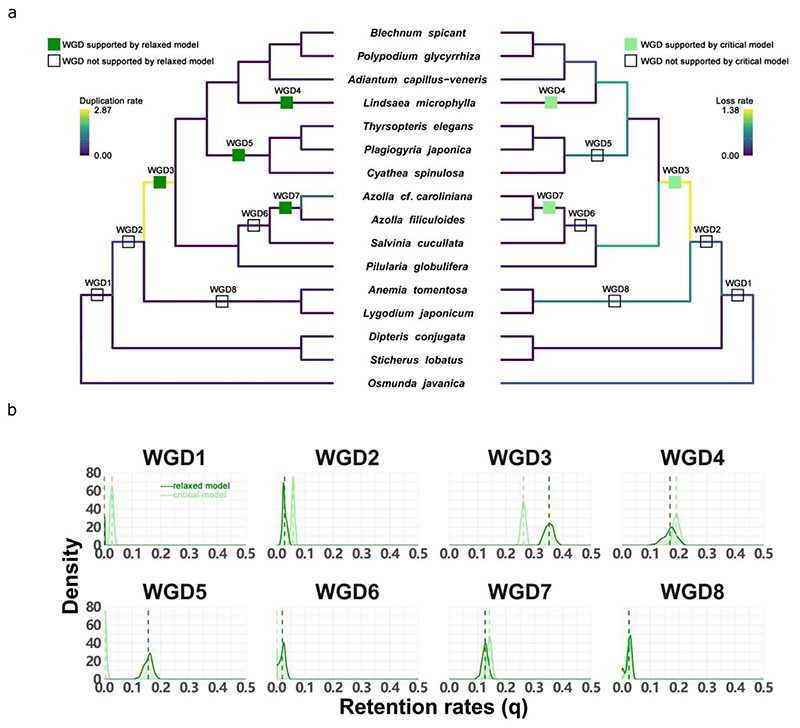
Whale (gene tree – species tree reconciliation) analysis for eight hypothetical WGDs under the DL+WGD model. (a) The species cladograms with the eight putative WGD events mentioned in the previous *K*_s_-age analyses. The WGD bars in green on the left cladogram (for the relaxed branch-specific model) and in light green on the right cladogram (for the critical branch-specific model) are supported WGDs with retention rates significantly different from zero, while the hollow WGD bars in each cladogram are the ones with retention rates not different from zero (Table 1). Posterior mean of duplication (left) and loss (right) rates estimated under the relaxed DL+WGD model (see Materials and Methods) are colored on the cladograms. (b) The posterior distributions of the WGD retention rates (*q*) for the eight putative WGDs under the relaxed branch-specific model (green) and the critical branch-specific model (light green). The dotted lines show the posterior mean of each posterior distribution.

To determine the exact positions of these potential WGDs, we used the so-called DL+WGD model implemented in Whale to perform statistical gene tree – species tree reconciliation (Zwaenepoel & Van de Peer, 2019), with inferred posterior probability distributions of gene trees for 6,863 gene families. To this end, the eight hypothetical WGDs, according to the *K*_s_ analyses, were placed on the species tree (Fig. 3; Fig. S6), each with a uniform prior for the WGD retention rate (*q*). Whale then used amalgamated likelihood estimation (szöllõsi *et al*., 2013) to test WGD hypotheses in a phylogenetic context through estimating duplication (*λ*) and loss (*μ*) rates for SSDs, and *q* for WGDs (Zwaenepoel & Van de Peer, 2019). Because assuming constant DL rates of SSDs across the species tree could substantially affect WGD testing (Zwaenepoel & Van de Peer, 2019), we adopted two models to incorporate various DL rates of SSDs across the species tree: 1) the critical branch-specific model, where each branch in the species tree has an equal DL rate, i.e., *λ* = *μ*, but the rates vary across branches; and 2) the relaxed branch-specific model, where DL rates again vary across branches but not necessarily equal, i.e., *λ* ≠ *μ*. Comparing results from different models may aid in assessing the robustness of particular inferences to model violations, because the basic linear birth-death process in the DL model may not be an ideal model of gene family evolution (Zwaenepoel & Van de Peer, 2021).

After obtaining posterior distributions of all the parameters under both DL+WGD models (Fig. 3), we estimated *q* for each putative WGD by its posterior mean. Further, we used the posterior distributions of *q* to estimate the Bayes Factor (*K*) to test if *q* is significantly different from zero using the Savage-Dickey density ratio (Zwaenepoel & Van de Peer, 2019). A putative WGD with *q* significantly larger than zero would hence indicate the occurrence of a WGD on a specific branch (Table 1). With the relaxed DL+WGD model, our results support four WGDs, i.e., WGD3, WGD4, WGD5, and WGD7, which all have $\bar{q}$ over 0.05. Similarly, the results based on the critical branch-specific DL+WGD model support WGD3, WGD4, and WGD7 (Fig. 3). Our gene tree – species tree reconciliation analyses with both DL+WGD models raised our confidence in resolving the two ambiguous WGDs discussed higher.

#### The *Azolla* WGD

**Fig. 4:**
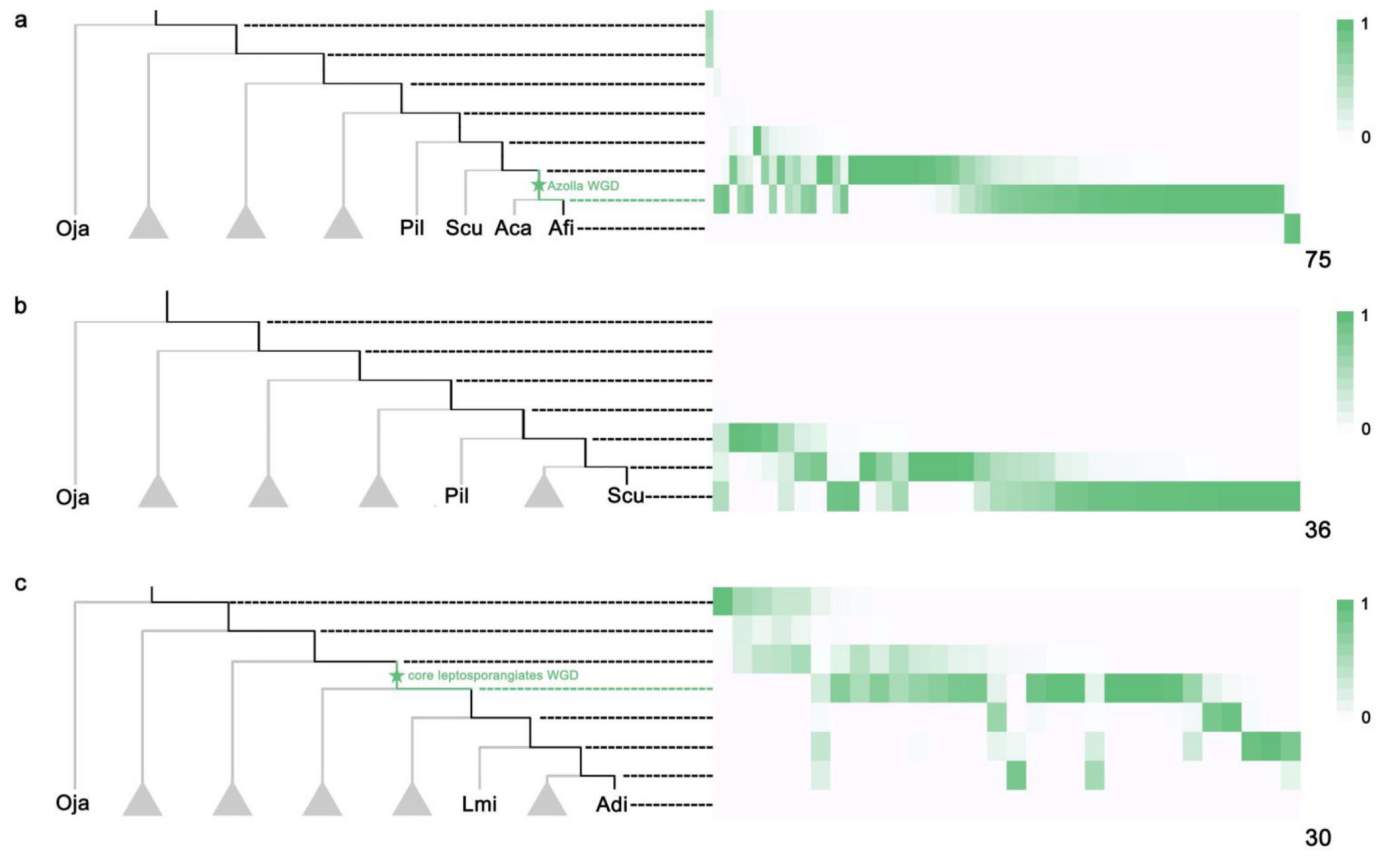
Gene tree - species tree reconciliation analyses for anchor pairs identified in the three fern genomes, *Azolla filiculoides, Salvinia cucullata*, and *Adiantum capillus-veneris*. On the phylogenetic trees, branches highlighted in black are the ones to which an anchor pair in the genomes of (a) *Azolla filiculoides*, (b) *Salvinia cucullata*, and (c) *Adiantum capillus-veneris* can be reconciled in the gene tree – species tree reconciliation analyses. Note that the reconciliation result of a pair of paralogs in Whale is not a duplication event on a specific branch but a posterior distribution over the possible branches in the species phylogeny on which the duplicate may reconcile to. On the right, the total number of columns in each heatmap, denoted at the right bottom corner, is the number of anchor pairs in analyzed. The squares in white to green in each column show the posterior probability that an anchor pair is reconciled as a duplication event to the respective branch. The color code ranges from white (posterior probability equal to zero) to green (posterior probability equal to one).

Both the relaxed and critical branch-specific models strongly support a WGD in the lineage leading to *Azolla* (WGD7) rather than a WGD shared by *Azolla* and *Salvinia* (WGD6) (Fig. 3; Table 1). Although the latter seems to have some support from the *K*_s_ analyses (Fig. 2d), the peak in *Azolla cf*. *caroliniana* is insignificant in the SiZer analysis (Fig. S3), and the peak in *Salvinia cucullata* may be artificial due to substitution saturation for its highest substitution rate among the analyzed species in Salviniales (Fig. 2b). Given the availability of *Azolla filiculoides* and *Salvinia cucullata* genomes (Li *et al*., 2018), we studied intra-genomic collinearity in each species and identified anchor pairs. Further, we examined the *K*_s_ distributions (Fig. S8) and the Whale reconciliation results for anchor pairs (Fig. 4). Except for some anchor pairs reconciled with high posterior probability to the species-specific branch, anchor pairs from the *Azolla filiculoides* genome tend to support the WGD specific to the *Azolla* genus (WGD7) rather than a WGD shared by *Azolla* and *Salvinia* (WGD6) (Fig. 4a). Also, the reconciliation results for anchor pairs from *Salvinia cucullata* only lend little support for a WGD shared by *Azolla* and *Salvinia* (Fig. 4b). In addition, a further inter-genomic collinearity comparison shows that the syntenic ratio of *Azolla filiculoides* : *Salvinia cucullata* : *Adiantum capillus-veneris* is 2 : 1 : 1, which again confirms our conclusion of one round of WGD experienced by *Azolla*, while no evidence for a WGD on the branch leading to *Adiantum* and *Salvinia* (Fig. S12).

#### The WGD shared by core leptosporangiates

With respect to WGD2 and WGD3, our Whale results support the WGD shared by core leptosporangiates (WGD3) but reject a WGD before the divergence between core leptosporangiates and Schizaeles (WGD2) (Fig. 3; Table 1). In the critical branch-specific model, the $\bar{q}$ for WGD2 is over 0.05 and larger than that in the relaxed branch-specific model. Also, although the Bayes Factor of WGD2 slightly favors *q* over zero, it cannot provide strong evidence for the occurrence of a WGD. By further examining the anchor pairs in the *A*. *capillus-veneris* genome (Fig. S8), we found that the Whale reconciliation results for the anchor pairs also only support a WGD shared by the core leptosporangiates (Fig. 4c).

#### One WGD in Cyatheales and one WGD in *Lindsaea*

The support of a WGD shared by Cyatheales is not as decisive as the one in the lineage leading to *Lindsaea*. The latter is supported by both DL+WGD models, as well as by the *K*_s_ analysis of *Lindsaea microphylla*. The former is, however, only supported by the *K*_s_ peak in *Cyathea spinulosa*, but not in the other two Cyathealean species. The critical branch-specific model has a low estimate for $\bar{q}$ ≈ 0, compared with $\bar{q}$ = 0.16 in the relaxed branch-specific model. In the relaxed model, the duplication rate is low ($\hat{\lambda }$ = 0.047), but the loss rate is high ($\hat{\mu }$= 0.551) on the branch leading to Cyatheales, so in the critical branch-specific model, the duplication rate is higher, whereas the loss rate is lower compared to the two rates estimated by the relaxed branch-specific model, respectively ($\hat{\lambda }$ = $\hat{\mu }$ = 0.313) (Fig. S6). Therefore, on the branch with WGD5, the assumption of equal DL rates in the critical branch-specific model appears to be strongly violated, although the rate differences in the branch-specific model appear to be unrealistic when interpreted as a model of gene family evolution. It seems prudent to conclude that support for WGD5 is not robust to model violations, and to abstain from further judgment based on these phylotranscriptomic analyses.

## Discussion

Accurate identification of WGDs in a phylogenetic context is the first and vital step to studying genome and chromosome evolution and the consequences of ancient polyploidy during evolution. Lacking high-quality genome assemblies, identifying WGDs in seed-free vascular plants has been primarily based on paranome *K*_s_-age distributions and gene tree – species tree reconciliations using transcriptome data. By revisiting both genomic and transcriptome data for leptosporangiate ferns, especially core leptosporangiates, we show that various synonymous substitution rates are present among the lineages of core leptosporangiates, suggesting that direct comparisons between WGD *K*_s_ peaks and speciation events under the assumption that lineages have similar substitution rates can be misleading. Therefore, considering various substitution rates across lineages is essential in correctly interpreting the identified *K*_s_ peaks.

In our analyses of the three species in Cyatheales, for instance, the *K*_s_ peak identified in *Cyathea spinulosa* supports a WGD shared by the three species, while the *K*_s_ peak identified in *Thyrsopteris elegans* supports a WGD shared by core leptosporangiates. In contrast, the 1KP initiative (2019), Huang et al. (2020), and Huang et al. (2022) all support a WGD in the lineage leading to *Cyathea* (‘CYATα’ and ‘11’ in Fig. 1) and a shared WGD for Cyatheales (‘CYATβ’ and ‘9’ in Fig. 1). In addition, according to Huang et al. (2020), there is another WGD in the lineage leading to *Plagiogyria* (‘10’ in Fig. 1). However, the *K*_s_ peaks identified in our analyses neither support a WGD in *Cyathea* (‘CYATα’ and ‘11’ in Fig. 1) or in *Plagiogyria* (‘10’). Evidently, the three species in Cyatheales have the lowest substitution rates among the core leptosporangiates (Fig. 2a). Therefore, the three studies above might have misinterpreted the peak in *Cyathea spinulosa* at *K*_s_ ≈ 0.3 as evidence for a recent WGD event in the lineage leading to *Cyathea*, while this peak is actually the result of a more ancient WGD with small *K*_s_ values due to the comparatively low substitution rates. Correspondingly, although Huang et al. (2022) specified consistent peaks (*K*_s_ > 1) in several Cyathealean species as the evidence for the Cyathealean WGD, those peaks are all in support of the WGD shared by core leptosporangiates.

Similarly, considering various substitution rates in Salviniales and Polypodiales also show placements of WGDs different from the 1KP initiative (2019) and Huang et al. (2020). In Salviniales, Huang et al. (2020) found no evidence for WGD, but our *K*_s_ analyses, along with the 1KP initiative (2019), and Li et al. (2018), identified one WGD in the lineage leading to *Azolla*. Within Polypodiales, both the 1KP initiative (2019) and Huang et al. (2020) identified two WGDs. One is placed in the lineage of *Lindsaea* and is supported by both studies (‘LINDα’ and ‘12’ in Fig. 1), as well as by our *K*_s_ analyses (Fig. 2d). Further, the 1KP initiative (2019) suggests that the other WGD (‘PTERα’ in Fig. 1) is shared by *Polypodium*, *Blechnum*, and *Adiantum*, whereas Huang et al. (2020) suggests that the WGD (‘3’ in Fig. 1) is only shared by *Polypodium* and *Blechnum*. However, the synonymous substitution rates in *Blechnum spicant* and *Polypodium glycyrrhiza* are lower than that in *Lindsaea microphylla* (Fig. 2c), suggesting that the *K*_s_ peaks identified in the two species may signal a more ancient WGD shared by core leptospongiates instead of WGD ‘PTERα’ or WGD ‘3’. Note that in each of the three orders of core leptosporangiates, there is at least one species that lends support for an ancient WGD shared by all the core leptosporangiates (or even before the divergence between Schizaeales and core leptosporangiates) as identified by the 1KP initiative (2019) (‘CYAT*γ*’ in Fig. 1). In contrast, Huang et al. (2020) have indicated a WGD shared by Polypodiales and Cyatheales (‘2’ in Fig. 1), which has no support from the 1KP initiative (2019), nor from our results.

Although analyses of *K*_s_-age distributions and considering different substitution rates across lineages could already reject some of the WGDs proposed in earlier studies (Fig. 2d), some *K*_s_ peaks from different species fall in competing branches adjacent to each other in the species phylogeny and remain therefore ambiguous. Two such cases are related to WGD2 and WGD3, and WGD6 and WGD7 (Fig. 3). As a signal to identify WGDs, the accuracy of estimating peak values in *K*_s_-age distributions can be affected by the WGD age and substitution rates. Indeed, taking a species with two WGDs as an example, the *K*_s_ peak for the recent WGD is often clear, but the *K*_s_ peak from the ancient WGD tends to be hidden in a flattened distribution and sensitive to distribution curve fluctuations due to different rates between paralogs or gene loss (or missing). Moreover, for species with high substitution rates, identifying peaks representing an ancient WGD at large *K*_s_ value may be confounded by substitution saturation to different extents (Vanneste *et al*., 2013), leading to less accurate estimates of *K*_s_ peak values. Also, both our rate correction approaches keep corrected rates as constants, while substitution rates likely have changed over time during evolution, leading to potentially inaccurate comparisons between *K*_s_ peaks and (especially ancient) speciation events. Therefore, solely relying on *K*_s_-age distributions is sometimes problematic, especially in species with high substitution rates.

Gene tree – species tree reconciliation is a complementary approach that, in some cases, can provide support for some very ancient WGDs (Jiao *et al*., 2011; Zwaenepoel & Van de Peer, 2019). Here, we adopted a statistical gene tree – species tree reconciliation method that considers gene tree uncertainties and other pitfalls known in LCA reconciliation. Unlike the DL+WGD model with an ML scheme, in which a series of likelihood ratio tests are performed by removing only one WGD at a time to test its likelihood of occurrence (Tiley *et al*., 2016), Whale adopts a Bayesian scheme to formally test the eight hypothetic WGDs, obtained by the *K*_s_ analyses (Fig. 3). For the four hypothetical WGDs on adjacent branches that could not be fully resolved by *K*_s_-age distributions, the Whale analyses with both DL+WGD models raised our confidence in WGD7 over WGD6, and WGD3 over WGD2. Also, the support of a WGD in the lineage leading to *Lindsaea* (WGD4) is clear. Nevertheless, the WGD shared by Cyatheales (WGD5) shows the necessity to assess the performance of the critical and relaxed branch-specific DL+WGD models, suggesting the need for more realistic models for genome evolutionary processes.

Here, we focused on the core leptosporangiates, because both our *K*_s_ and reconciliation analyses required suitable outgroups. Beyond the core leptosporangiates, the *K*_s_ peak identified in *Anemia tomentosa* indicates a shared WGD with *Lygodium japonicum*. However, the *K*_s_ peak in *Lygodium japonicum* goes against a shared WGD with *Anemia tomentosa* but suggests a more ancient WGD, which is also supported by the *K*_s_ peaks in *Dipteris conjugata* and *Sticherus lobatus* (Fig. 2d). In general, our results seem to largely agree with the 1KP initiative (2019), which identified a WGD in *Anemia tomentosa* (‘LYGOα’ in Fig. 1), but no WGDs in *Dipteris* and *Sticherus*, in contrast to those in Huang et al. (2020) (‘7’ and ‘8’ in Fig. 1). For the more ancient WGDs identified by both previous studies (‘OSMNβ’, ‘HYMEα’, and ‘1’ in Fig. 1), our analyses could not resolve whether a WGD occurred before the divergence of leptosporangiates (‘OSMNβ’ in Fig. 1) and/or a WGD occurred after *Osmunda javanica* diverged from the rest of leptosporangiates (‘HYMEα’ and ‘1’ in Fig. 1), because *Osmunda javanica* is an outgroup in the phylogenetic tree (Fig. 2d). Without extra information we cannot determine when *Osmunda javanica* diverged from other leptosporangiates. This is also why, although there is a *K*_s_ peak in the distribution for *Osmunda javanica* (Fig. S3), we were uncertain about assuming a WGD either shared with other leptosporangiates or a species-specific WGD (‘OSMNα’ and ‘14’ in Fig. 1). Likewise, our Whale analyses show no support for WGD1 and WGD8 (Fig. 3), as they were placed in the outgroups of core leptosporangiates, so the species sampling may be less suitable to resolve these WGDs. For example, WGD1 may be the result of two WGDs that have occurred on two consecutive branches, if we accept the results from the 1KP initiative (2019). Without species that can further break down the branch in the species phylogeny where WGD1 is located, it is difficult to neatly solve the problem with either the *K*_s_-age or the reconciliation approaches (Zwaenepoel & Van de Peer, 2019). Although we could add extra species to determine the root or further break down long branches, this may introduce another species with a WGD event that cannot be resolved with certainty, so here we decided to only focus on the analyses in the core leptosporangiates.

In conclusion, neglecting differences in substitution rates and performing LCA reconciliations could lead to both false positives and false negatives in calling WGDs. Therefore, we underscore the importance of careful analysis, including the consideration of differences in substitution rates and appreciation of gene tree – species tree reconciliation uncertainties, prompting that failure to do so is likely to lead to unreliable or incorrect conclusions. In addition, we highlight the importance of developing better and more robust statistical models for genome evolutionary processes if we are to reliably characterize the evolutionary history of species at the genomic level.

## Supplementary Material

Figures, Tables, Methods

## Figures and Tables

**Table 1:** Hypothetical WGDs, posterior mean of duplicate retention rate (*q*), and the Bayes Factor (*K*) to compare the likelihood of *q* = 0 (H_0_) to the likelihood of *q* > 0 (H_1_) using the Savage-Dickey density ratio.

Hypotheses	Relaxed branch-specific model		Critical branch-specific model	
	$\bar{q}$	*K*	$\bar{q}$	*K*
WGD1	0.001	1272.297	0.029	2.693
WGD2	0.028	2.649	0.058	0.393
WGD3	0.352	0.047**	0.263	0.061**
WGD4	0.170	0.197*	0.193	0.094**
WGD5	0.156	0.149*	0.006	128.480
WGD6	0.020	21.026	0.000	6919.539
WGD7	0.127	0.166*	0.142	0.135*
WGD8	0.025	12.864	0.027	6.007

** *K* < 1/10 or *K* < 0.1, strong evidence against H_0_;

* *K* < 1/10^0.5^ or *K* < 0. 3162 substantial evidence against H_0_;

*K* < 1, H_1_ supported, not worth more than a bare mention; *K* > 1, H_0_ supported.

## Data Availability

The data that support the findings of this study are openly available as summarized in Table S1.
