# Supplementary material for: Revisiting Ancient Polyploidy in Leptosporangiate Ferns
Source: New Phytol. Author manuscript; Available in PMC 2023 Feb 1. (PMC7614084; doi:10.1111/nph.18607)
Supplement: Figures, Tables, Methods [file EMS157432-supplement-Figures__Tables__Methods.pdf]

New Phytologist

***New Phytologist* Supporting Information**

Article title: Revisiting Ancient Polyploidy in Leptosporangiate Ferns
Authors: Hengchi Chen, Yuhan Fang, Arthur Zwaenepoel, Sanwen Huang, Yves Van de Peer, Zhen Li
Article acceptance date: 30 October 2022

The following Supporting Information is available for this article:

Fig. S1 The number of genes in the transcriptome assemblies from the 1KP initiative (2019) and Huang et al. (2020).

Fig. S2 BUSCO analysis for the transcriptome assemblies from the 1KP initiative (2019).

Fig. S3 K_S distributions for the whole paranomes in different species with the Gaussian Mixture Modeling (GMM) analysis and the SiZer analysis.

Fig. S4 The Bayesian Information Criterion (BIC) score in the Gaussian mixture modeling analysis for different species in Fig. S3.

Fig. S5 The analyses of ksrates for different species.

Fig. S6 The time-calibrated species trees from TimeTree.

Fig. S7 The minimum effective sample size of tree length and the average standard deviation of split frequencies for the 1,000 randomly selected gene families.

Fig. S8 K_S distributions for anchor pairs identified in *Azolla filiculoides*, *Salvinia cucullata*, and *Adiantum capillus-veneris*.

Fig. S9 Box plots of the number of genes without tandem duplicates on scaffolds having anchor pairs with K_S values less than 0.1 and those having anchor pairs with K_S values near a potential WGD peak in the three fern genomes.

Fig. S10 The one-to-one orthologous K_S age distributions between *Dipteris conjugata* and species from Cyatheales, Salviniales, and Polypodiales.

Fig. S11 The K_S distribution for paranomes of *Thyrsopteris elegans* (upper) and *Plagiogyria japonica* (lower) within a K_S range of [0, 1.0] and a binwidth of 0.05.

Fig. S12 Ratios of collinear blocks for pairwise intergenomic comparisons among the three

genome-available ferns.

Table S1 Taxonomy, number of genes/unigenes and data source of fern species involved in this study.

Table S2 The mean, standard deviation (SD), Monte Carlo standard error (MCSE), effective sample size (ESS), and 95% uncertainty interval for parameters estimated under the critical branch-specific DL+WGD model.

Table S3 The mean, standard deviation (SD), Monte Carlo standard error (MCSE), effective sample size (ESS), and 95% uncertainty interval for parameters estimated under the relaxed branch-specific DL+WGD model.

Table S4 The mean, standard deviation (SD), Monte Carlo standard error (MCSE), effective sample size (ESS), and 95% uncertainty interval for parameters estimated under the critical branch-specific DL+WGD model for the randomly selected gene families.

Table S5 The mean, standard deviation (SD), Monte Carlo standard error (MCSE), effective sample size (ESS), and 95% uncertainty interval for parameters estimated under the relaxed branch-specific DL+WGD model for the randomly selected gene families.

Methods S1 Julia code for the Whale analyses with the critical and relaxed branch-specific DL+WGD models.

Methods S2 Julia code for the Whale analysis of gene tree – species tree reconciliations.

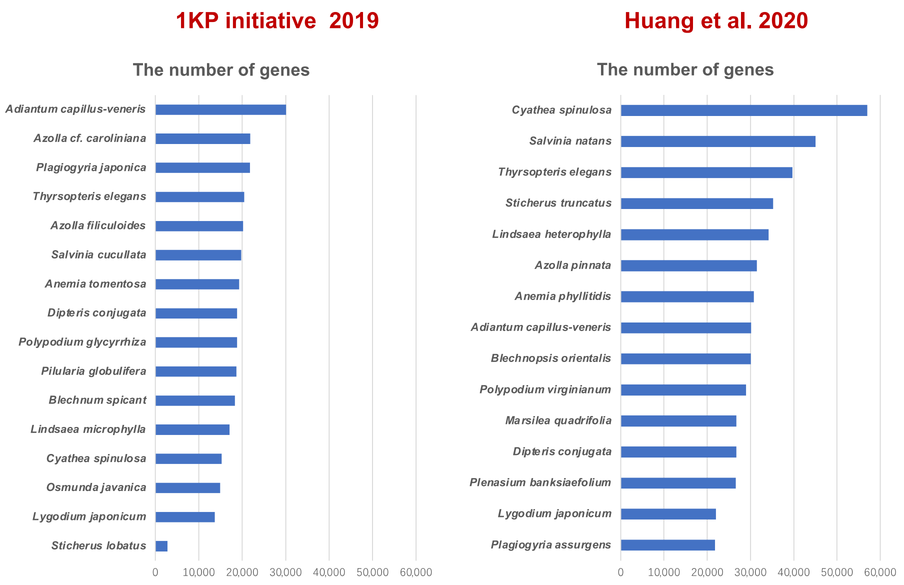

Fig. S1 The number of genes in the transcriptome assemblies from the 1KP initiative (2019) and Huang et al. (2020).

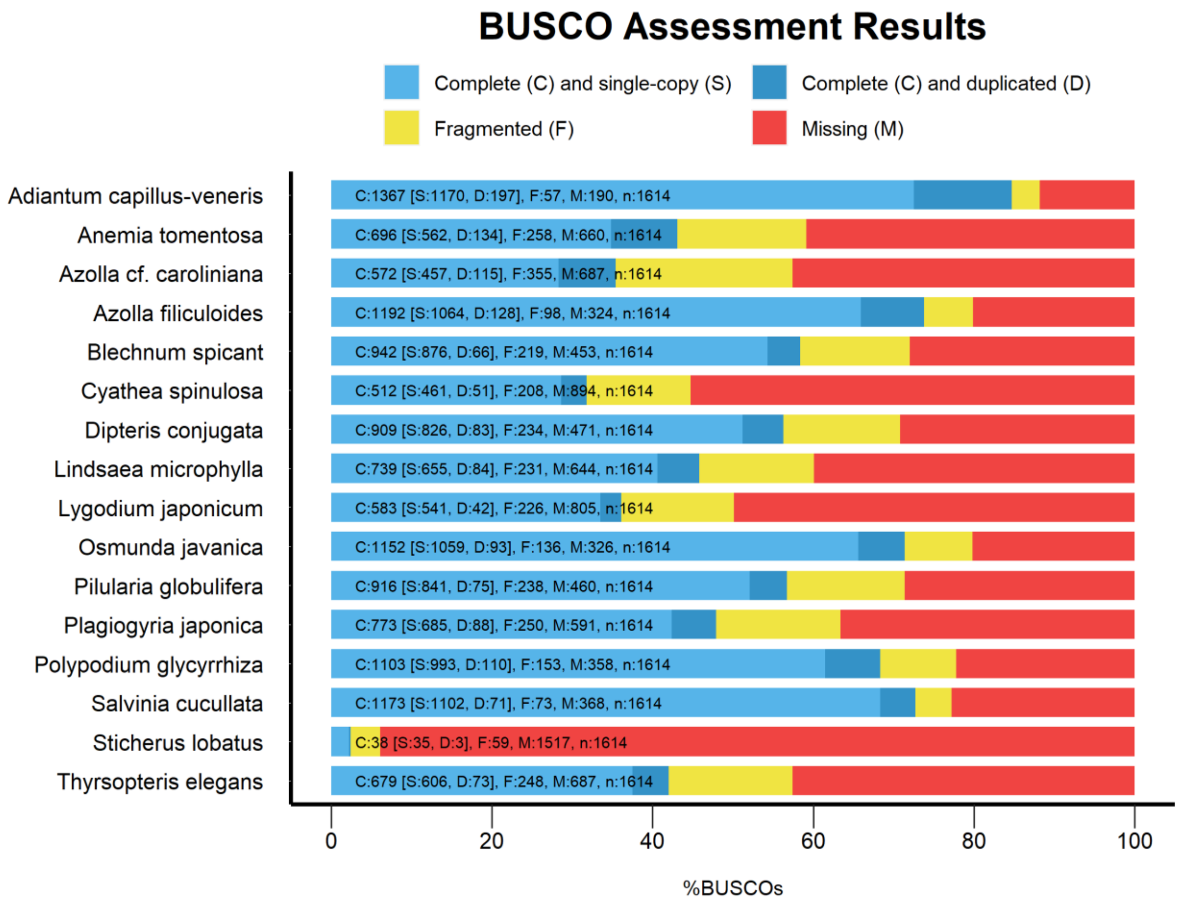

Fig. S2 BUSCO analysis for the transcriptome assemblies from the 1KP initiative (2019).

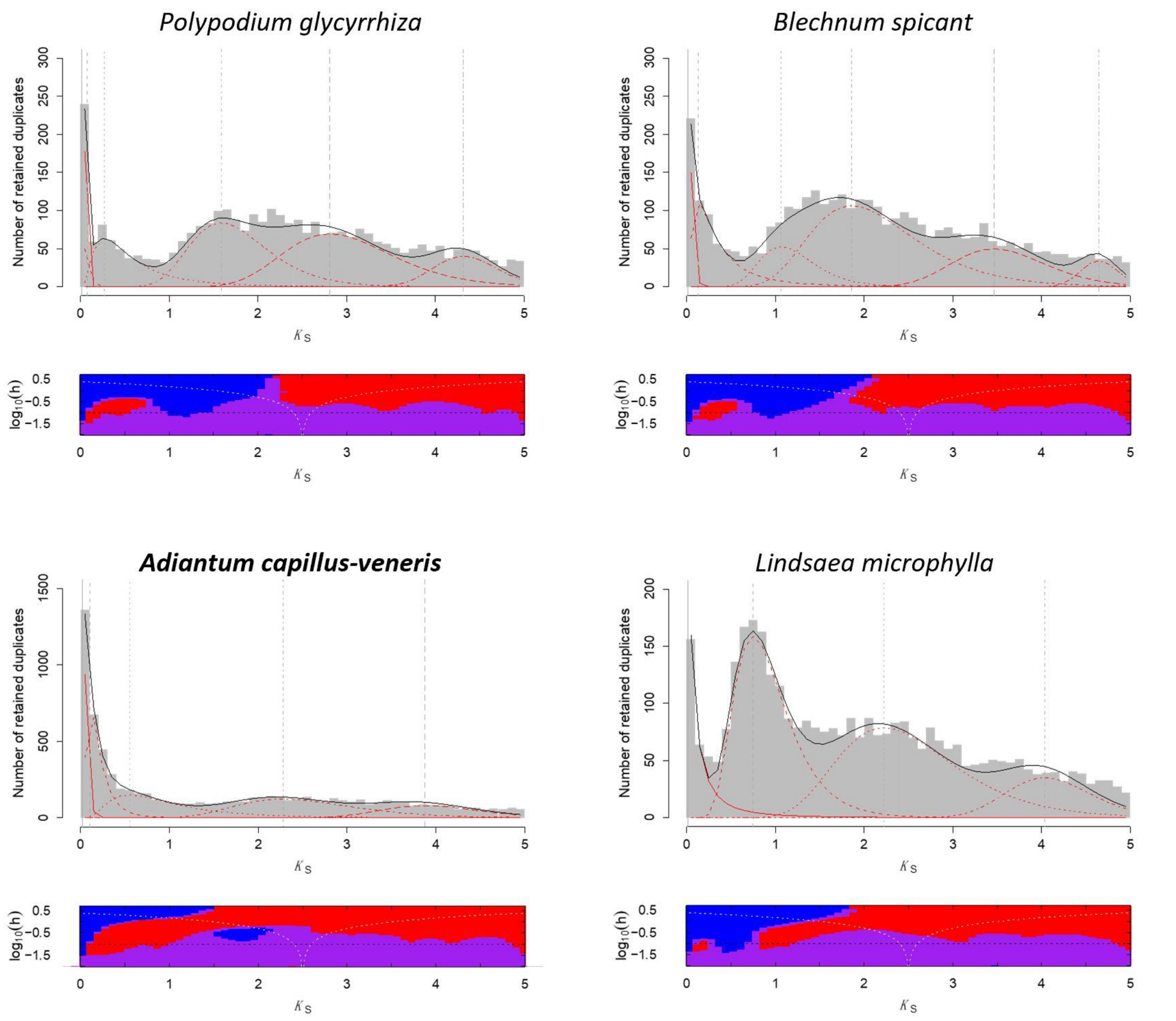

Fig. S3 K_S distributions for the whole paranomes in different species with the Gaussian Mixture Modeling (GMM) analysis and the SiZer analysis. The optimal number of log-normal components overlaid on K_S distributions in red curves with grey vertical lines representing modes, and black curves show the sum of components. In a SiZer slope plot, putative true peaks are enclosed by a blue stretch (significant upward slope) to the left and a red stretch (significant downward slope) to the right. Purple stretches correspond to no significant upward or downward slope, and gray stretches indicate regions where data is too sparse.

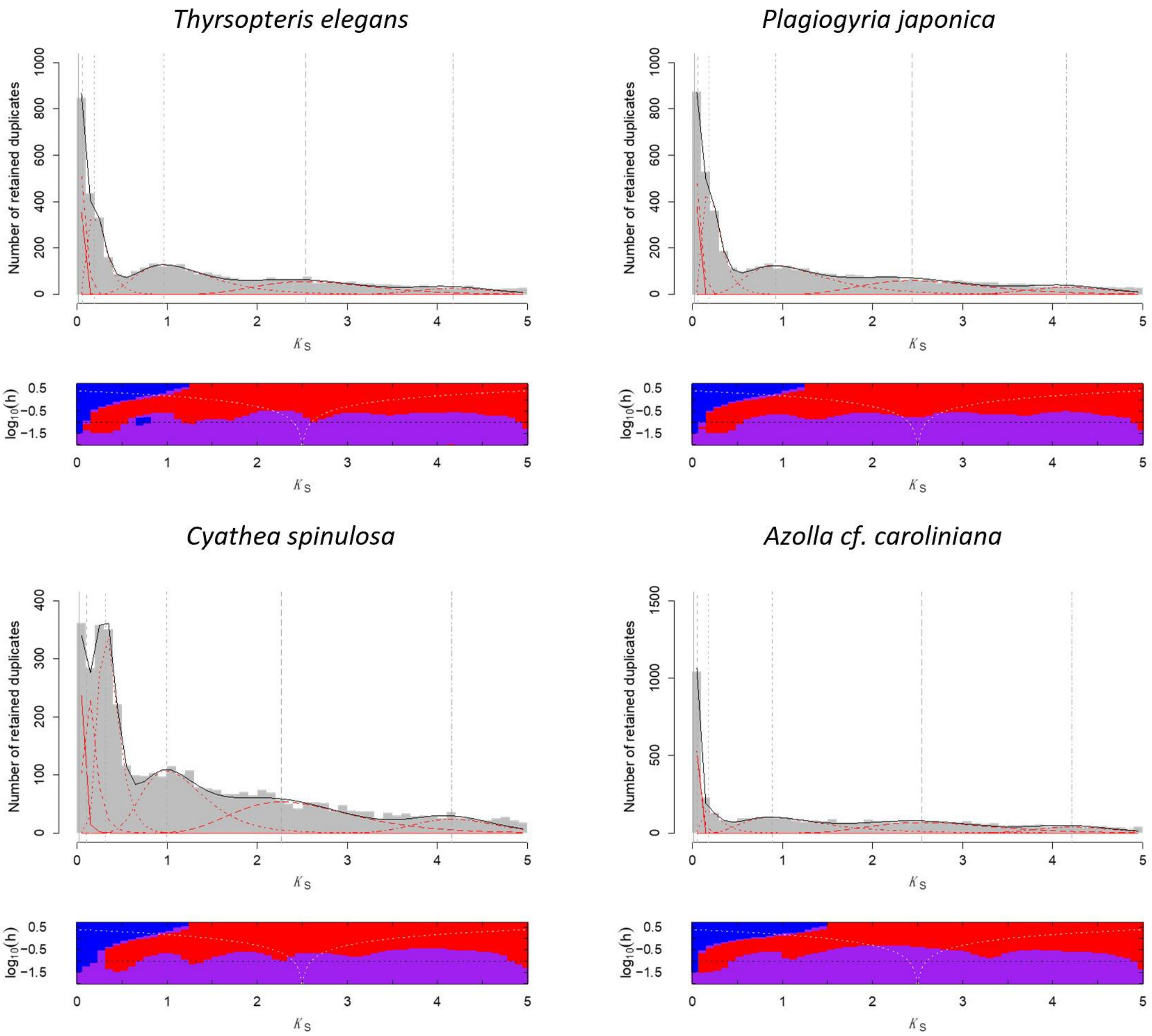

Fig. S3 (continued) K_S distributions for the whole paranomes in different species with the Gaussian Mixture Modeling (GMM) analysis and the SiZer analysis. The optimal number of log-normal components overlaid on K_S distributions in red curves with grey vertical lines representing modes, and black curves show the sum of components. In a SiZer slope plot, putative true peaks are enclosed by a blue stretch (significant upward slope) to the left and a red stretch (significant downward slope) to the right. Purple stretches correspond to no significant upward or downward slope, and gray stretches indicate regions where data is too sparse.

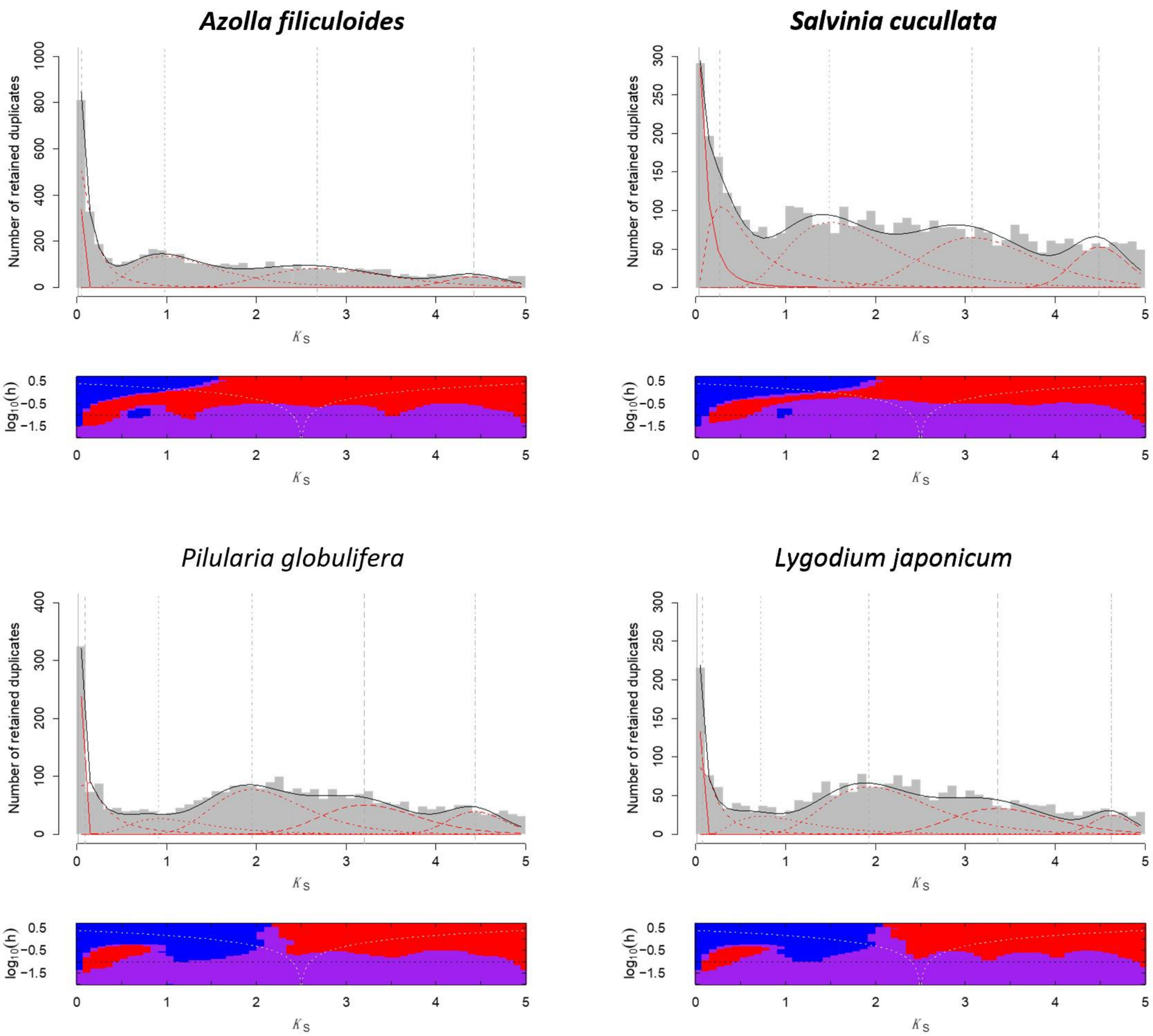

Fig. S3 (continued) K_S distributions for the whole paranomes in different species with the Gaussian Mixture Modeling (GMM) analysis and the SiZer analysis. The optimal number of log-normal components overlaid on K_S distributions in red curves with grey vertical lines representing modes, and black curves show the sum of components. In a SiZer slope plot, putative true peaks are enclosed by a blue stretch (significant upward slope) to the left and a red stretch (significant downward slope) to the right. Purple stretches correspond to no significant upward or downward slope, and gray stretches indicate regions where data is too sparse.

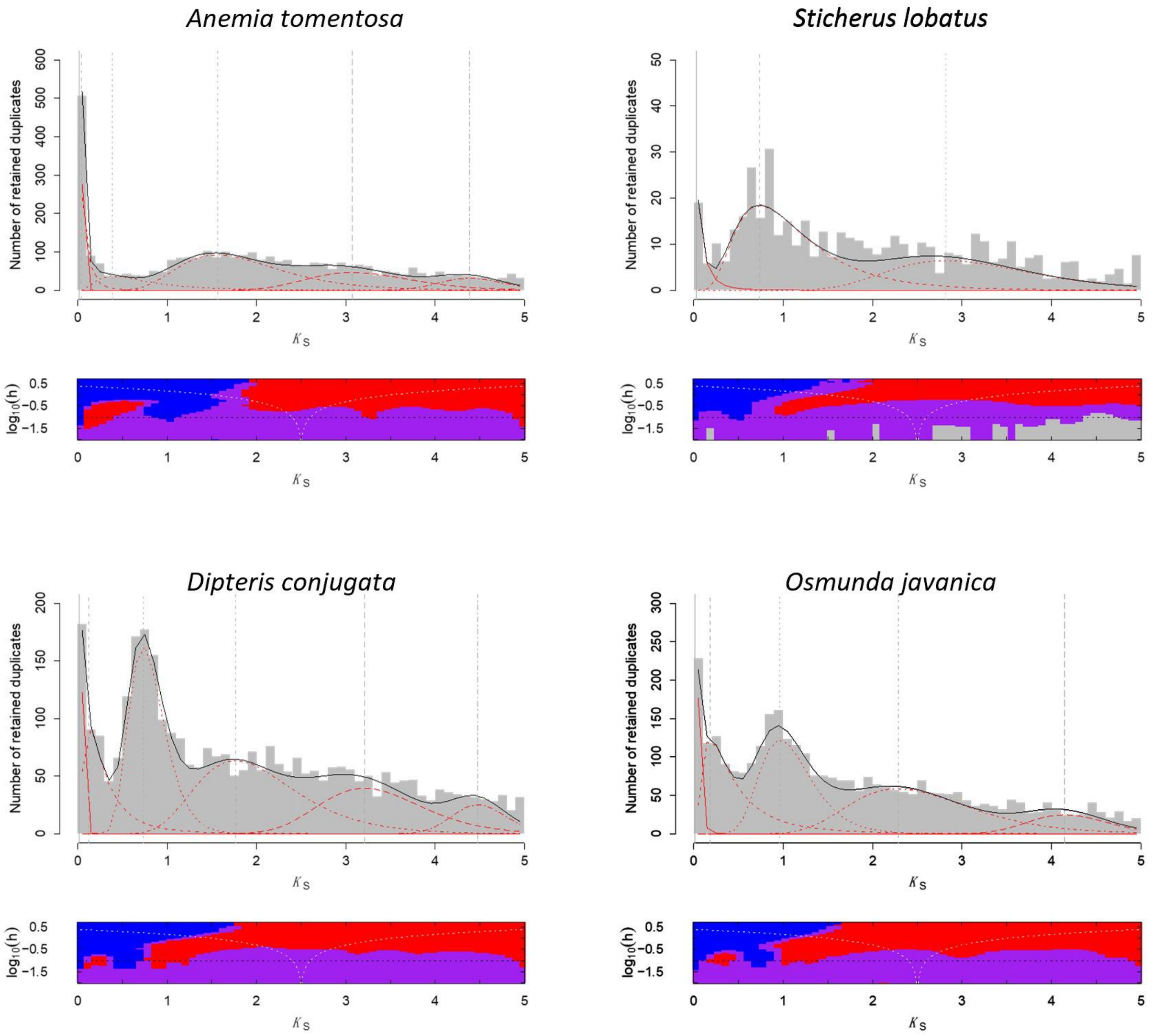

Fig. S3 (continued) K_S distributions for the whole paranomes in different species with the Gaussian Mixture Modeling (GMM) analysis and the SiZer analysis. The optimal number of log-normal components overlaid on K_S distributions in red curves with grey vertical lines representing modes, and black curves show the sum of components. In a SiZer slope plot, putative true peaks are enclosed by a blue stretch (significant upward slope) to the left and a red stretch (significant downward slope) to the right. Purple stretches correspond to no significant upward or downward slope, and gray stretches indicate regions where data is too sparse.

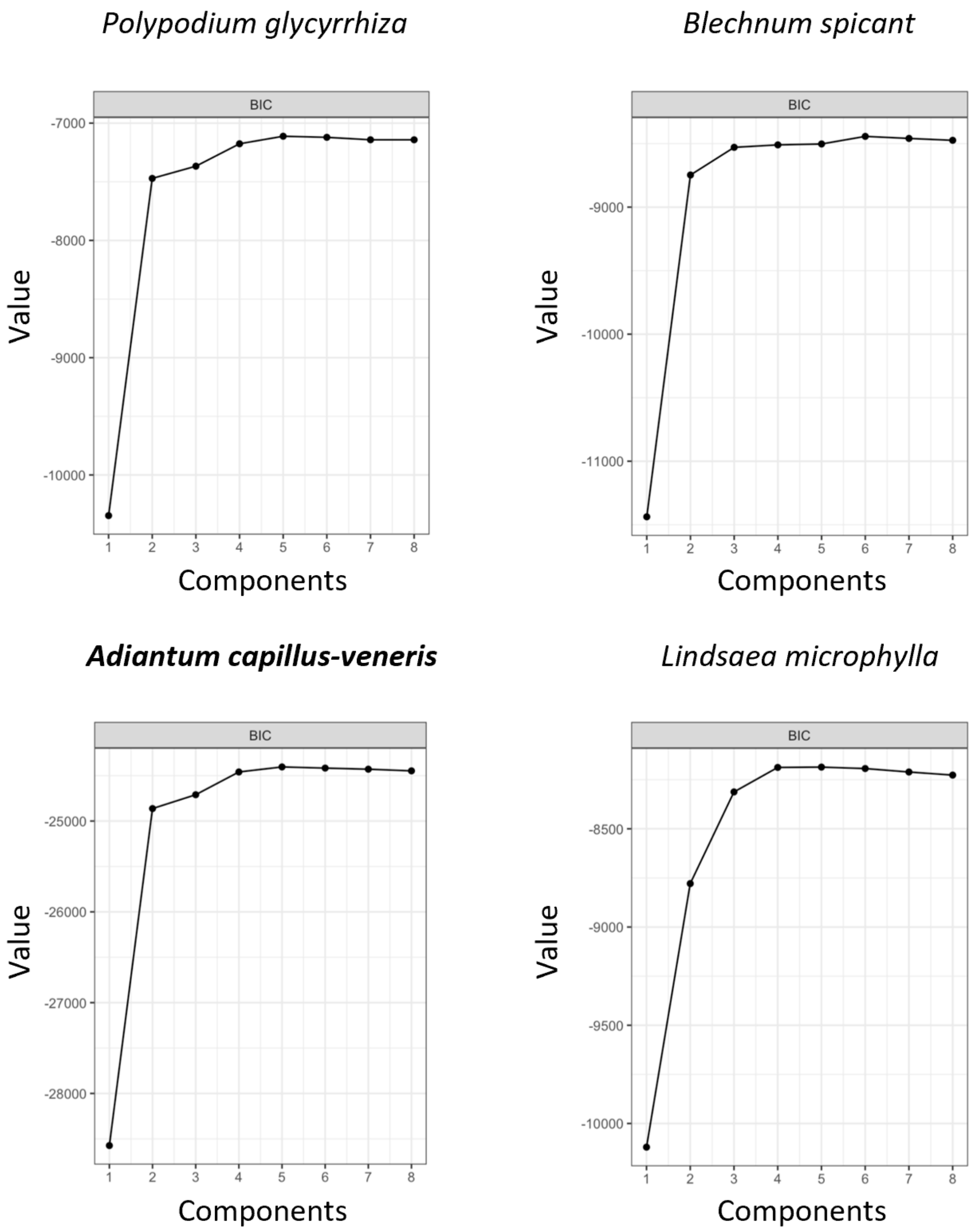

Fig. S4 The Bayesian Information Criterion (BIC) score in the Gaussian mixture modeling analysis for different species in Fig. S3.

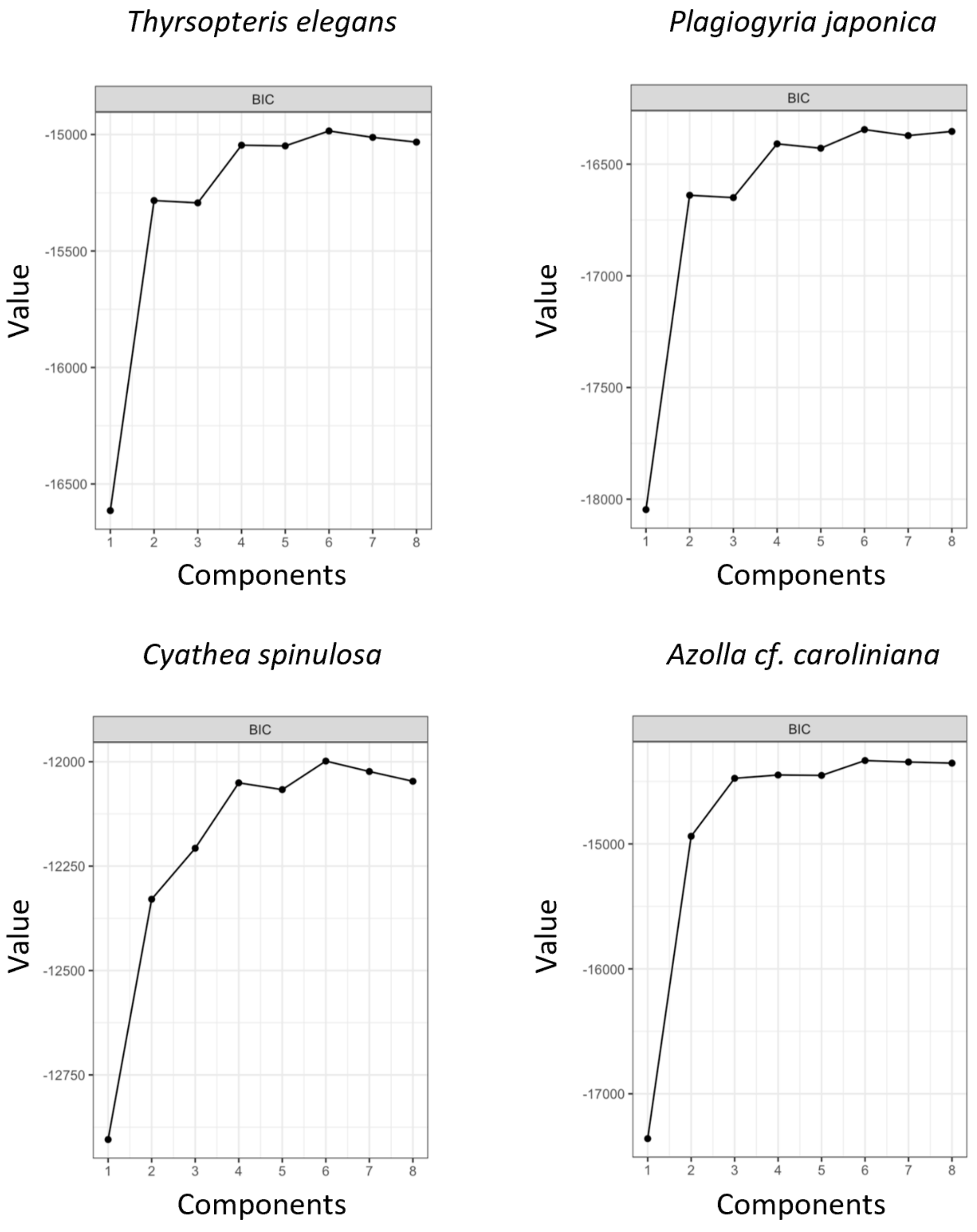

Fig. S4 (continued) The Bayesian Information Criterion (BIC) score in the Gaussian mixture modeling analysis for different species in Fig. S3.

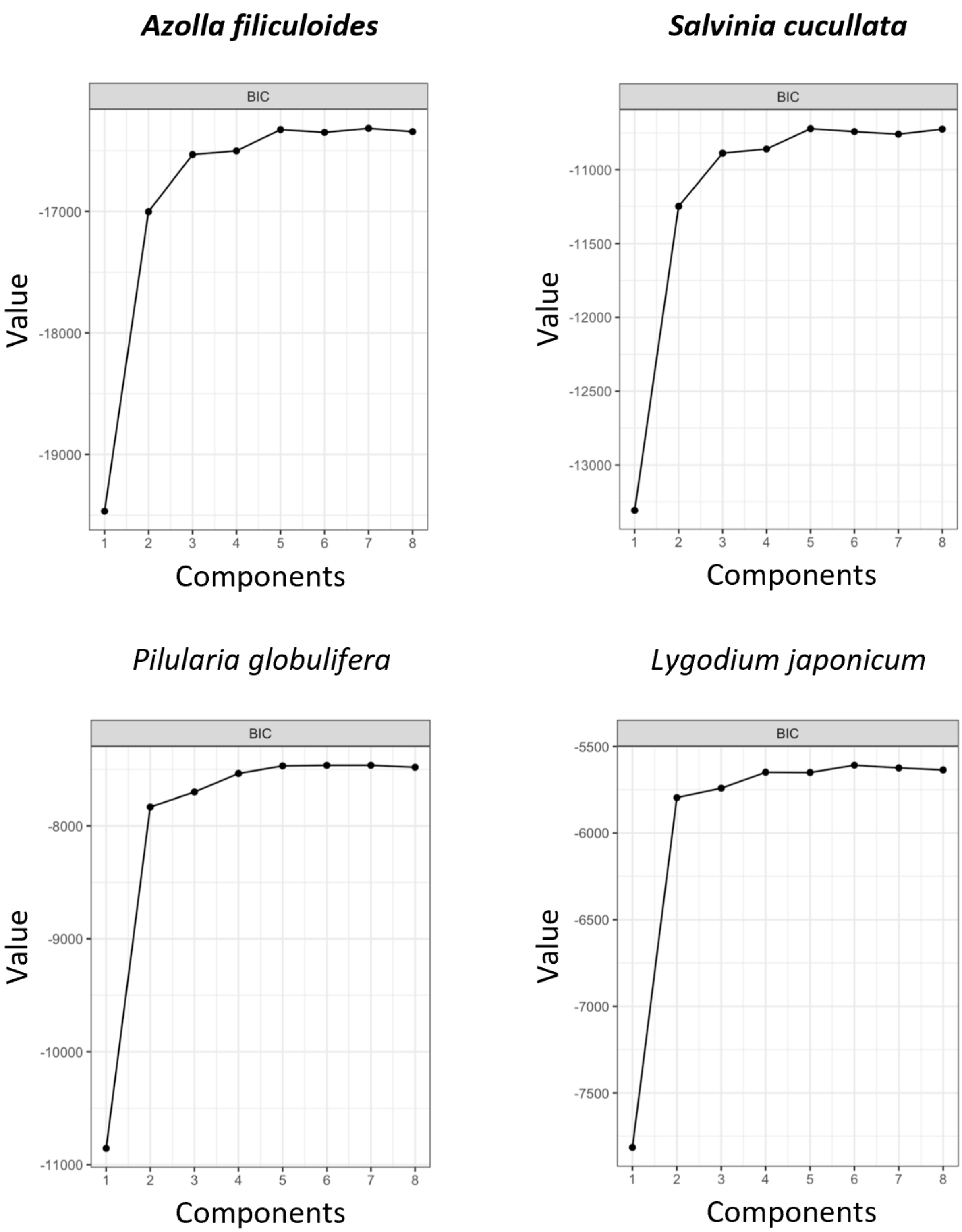

Fig. S4 (continued) The Bayesian Information Criterion (BIC) score in the Gaussian mixture modeling analysis for different species in Fig. S3.

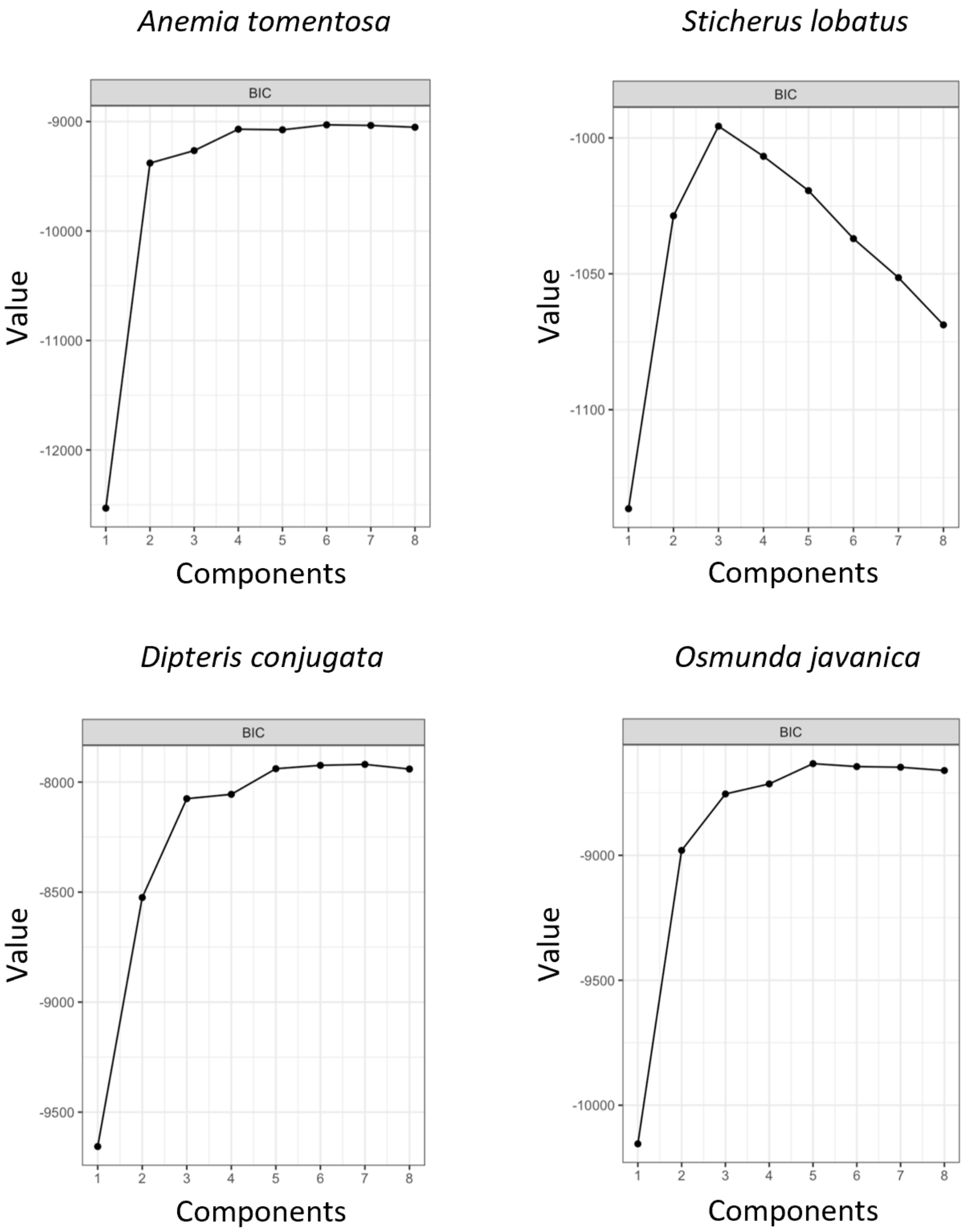

Fig. S4 (continued) The Bayesian Information Criterion (BIC) score in the Gaussian mixture modeling analysis for different species in Fig. S3.

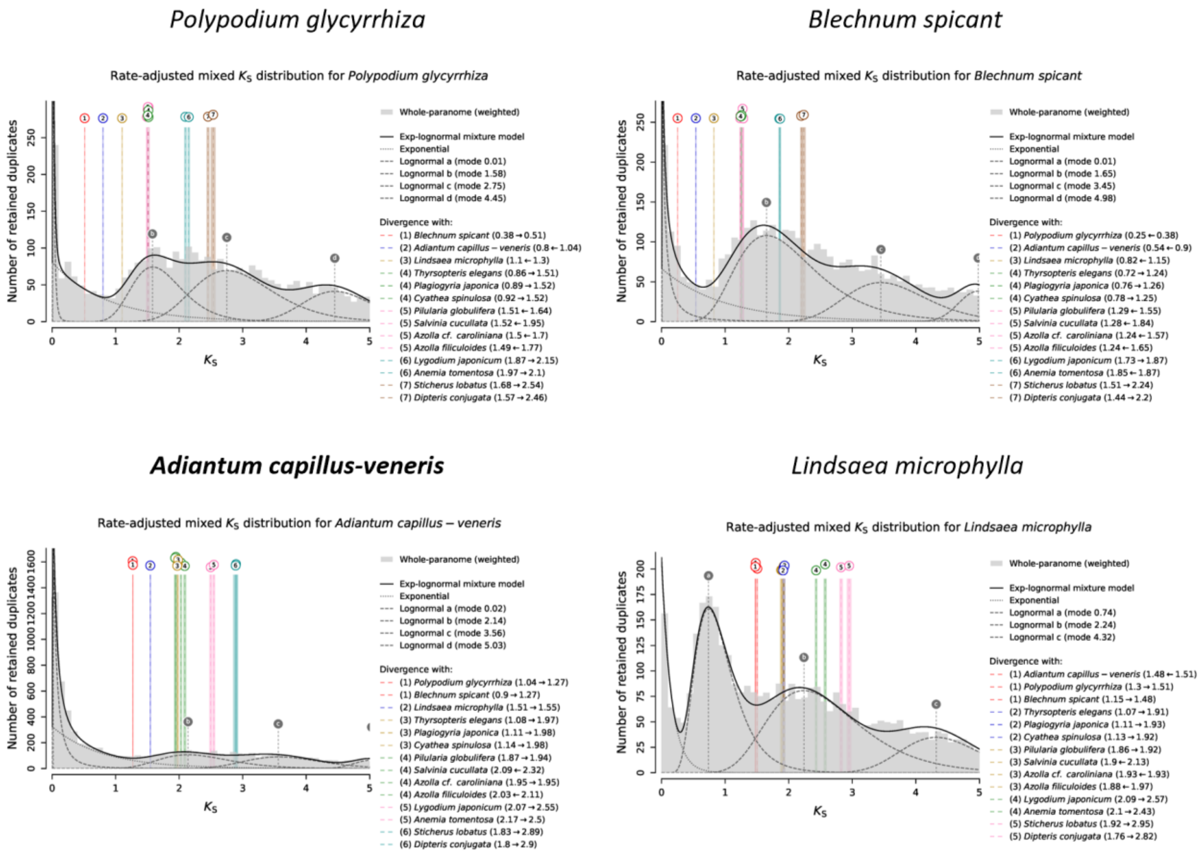

Fig. S5 The analyses of ksrates for different species. K_S distributions for the whole paranomes of different species are overlaid with rate-adjusted species events in colored vertical lines. The overall mixture model in the dark solid line of each paralogous K_S distribution consists of an exponential component in dotted gray curve and optimized log-normal components in dashed gray curves. Each log-normal component is labeled with a letter, shown as vertical dashed gray lines with circular labels. Rate-adjusted mode estimates of orthologous K_S distributions between a focal species and other species, representing speciation events, are drawn as numbered vertical long-dashed lines, with associated colored boxes showing the standard deviation and the mean of estimated mode. Lines representing the same speciation event in the phylogeny share color and numbering. Horizontal arrows in figure legends indicate the K_S shifts produced by the substitution rate adjustments.

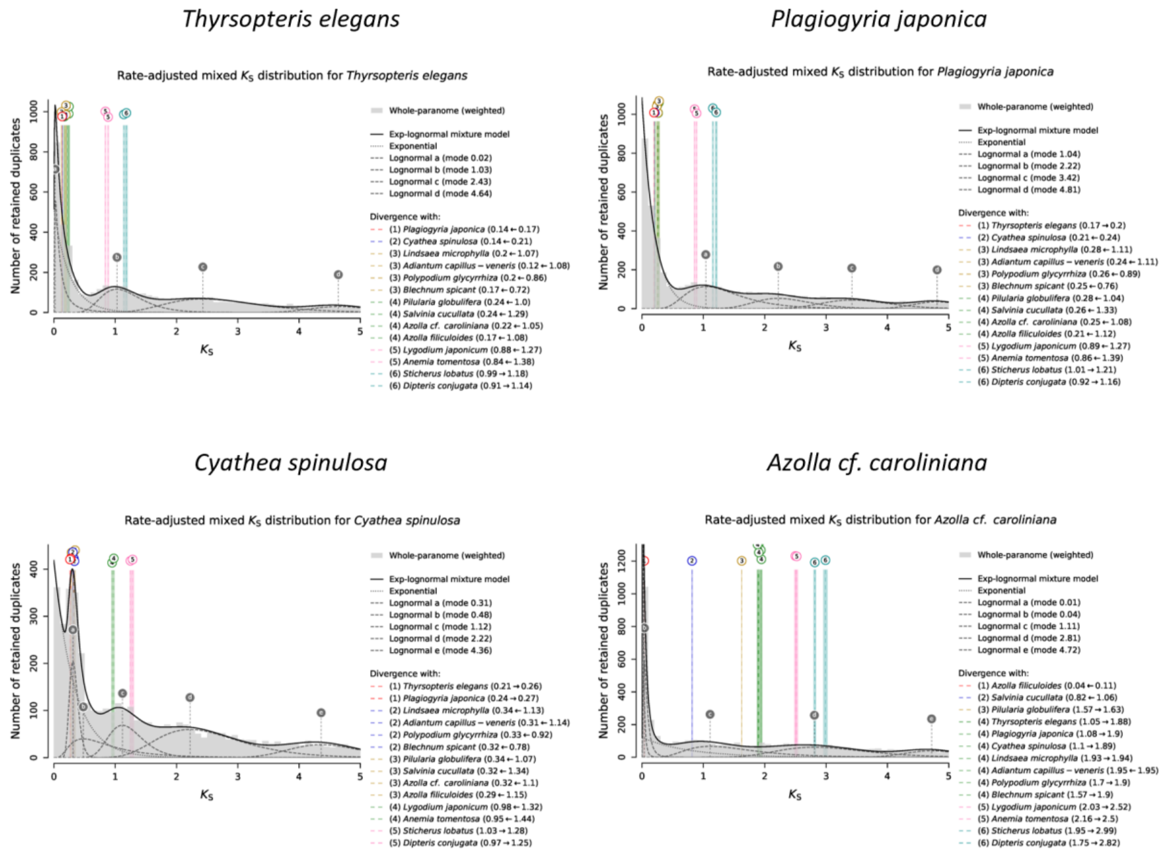

Fig. S5 (continued) The analyses of ksrates for different species. K_S distributions for the whole paranomes of different species are overlaid with rate-adjusted species events in colored vertical lines. The overall mixture model in the dark solid line of each paralogous K_S distribution consists of an exponential component in dotted gray curve and optimized log-normal components in dashed gray curves. Each log-normal component is labeled with a letter, shown as vertical dashed gray lines with circular labels. Rate-adjusted mode estimates of orthologous K_S distributions between a focal species and other species, representing speciation events, are drawn as numbered vertical long-dashed lines, with associated colored boxes showing the standard deviation and the mean of estimated mode. Lines representing the same speciation event in the phylogeny share color and numbering. Horizontal arrows in figure legends indicate the K_S shifts produced by the substitution rate adjustments.

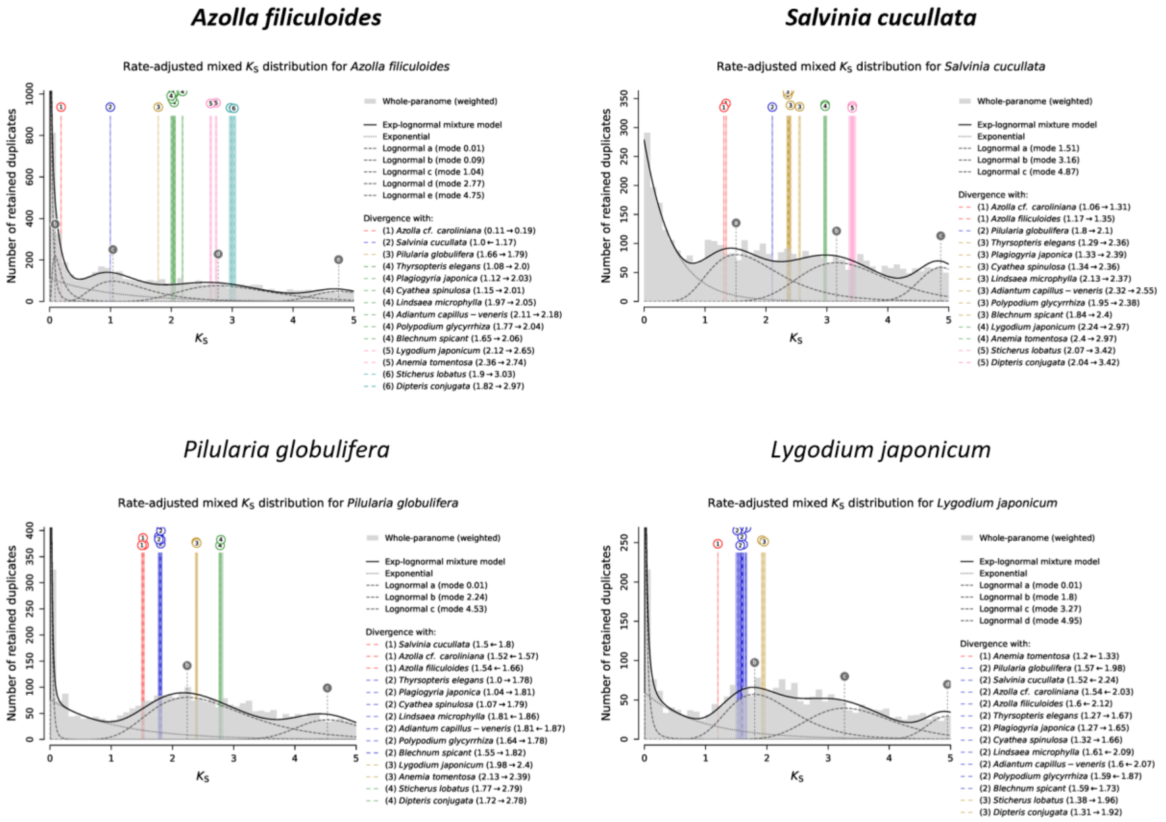

Fig. S5 (continued) The analyses of ksrates for different species. K_S distributions for the whole paranomes of different species are overlaid with rate-adjusted species events in colored vertical lines. The overall mixture model in the dark solid line of each paralogous K_S distribution consists of an exponential component in dotted gray curve and optimized log-normal components in dashed gray curves. Each log-normal component is labeled with a letter, shown as vertical dashed gray lines with circular labels. Rate-adjusted mode estimates of orthologous K_S distributions between a focal species and other species, representing speciation events, are drawn as numbered vertical long-dashed lines, with associated colored boxes showing the standard deviation and the mean of estimated mode. Lines representing the same speciation event in the phylogeny share color and numbering. Horizontal arrows in figure legends indicate the K_S shifts produced by the substitution rate adjustments.

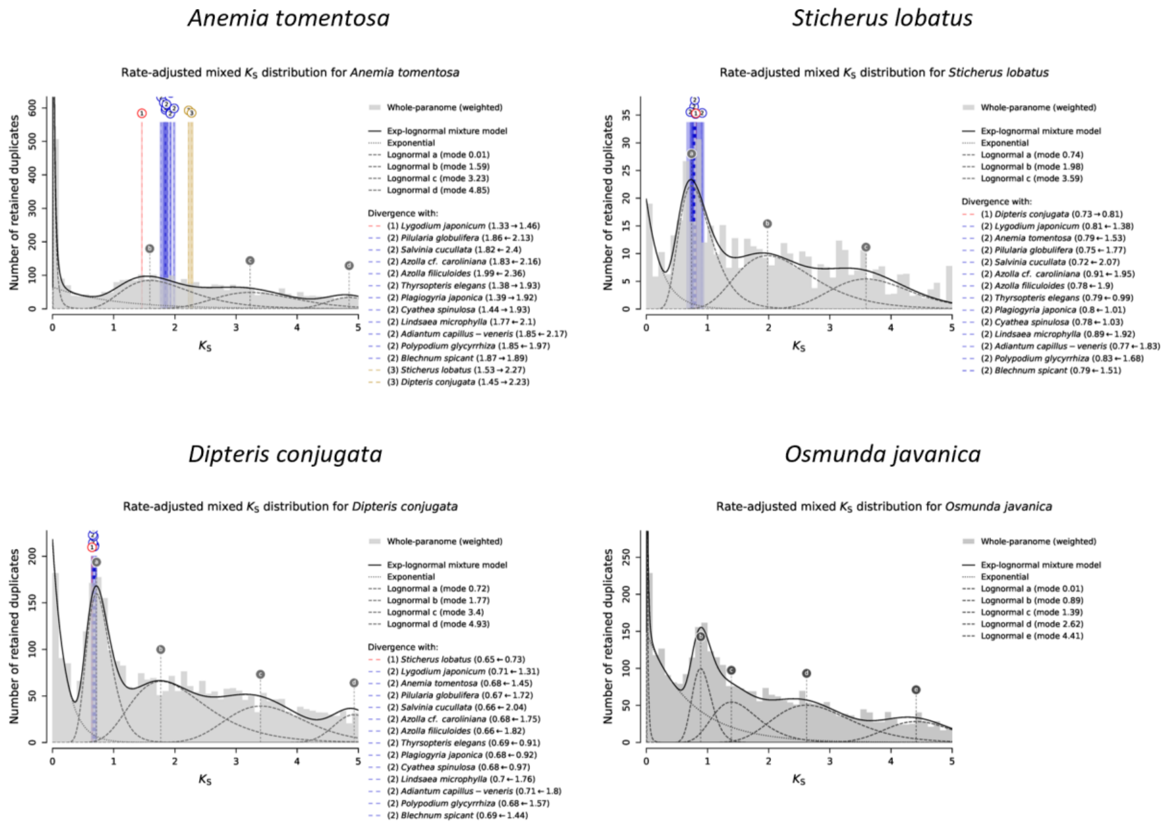

Fig. S5 (continued) The analyses of ksrates for different species. K_S distributions for the whole paranomes of different species are overlaid with rate-adjusted species events in colored vertical lines. The overall mixture model in the dark solid line of each paralogous K_S distribution consists of an exponential component in dotted gray curve and optimized log-normal components in dashed gray curves. Each log-normal component is labeled with a letter, shown as vertical dashed gray lines with circular labels. Rate-adjusted mode estimates of orthologous K_S distributions between a focal species and other species, representing speciation events, are drawn as numbered vertical long-dashed lines, with associated colored boxes showing the standard deviation and the mean of estimated mode. Lines representing the same speciation event in the phylogeny share color and numbering. Horizontal arrows in figure legends indicate the K_S shifts produced by the substitution rate adjustments.

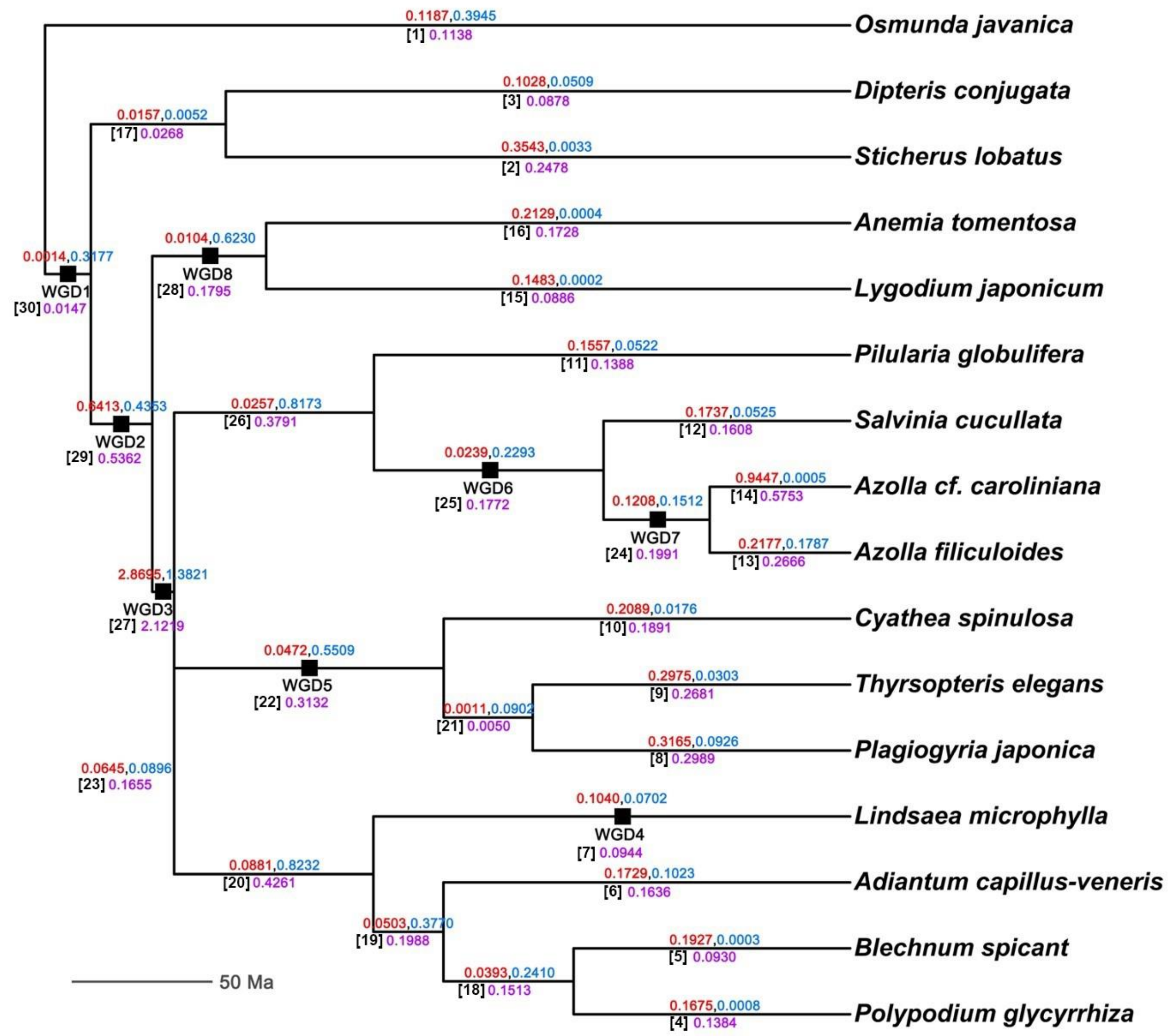

Fig. S6 The time-calibrated species trees from TimeTree (http://www.timetree.org). The numbers above a branch are the duplication (red) and loss (blue) rates in the relaxed branch-specific model. The number below a branch is the duplication and loss rates (purple), which are equal in the critical branch-specific model. The black squares on branches are the eight WGDs that were tested in the DL+WGD model. The numbers in brackets are the branch indices in the gene tree – species tree reconciliations.

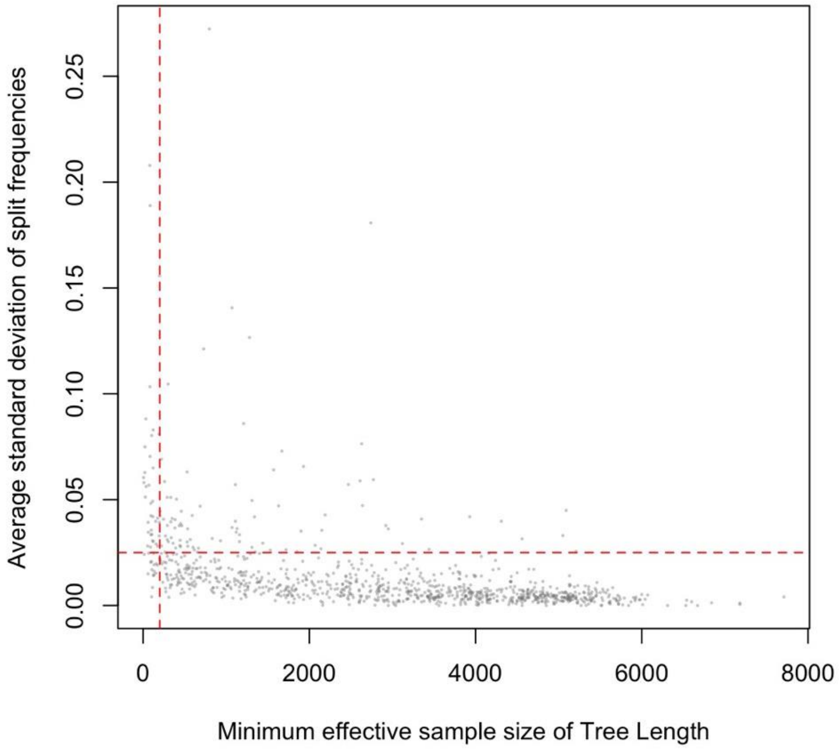

Fig. S7 The minimum effective sample size of Tree Length and the average standard deviation of split frequencies for the 1,000 randomly selected gene families. The gene families with the minimum effective sample size of tree length > 200 (the red dashed line in vertical) and the average standard deviation of split frequencies < 0.025 (the red dashed line in horizontal) were selected.

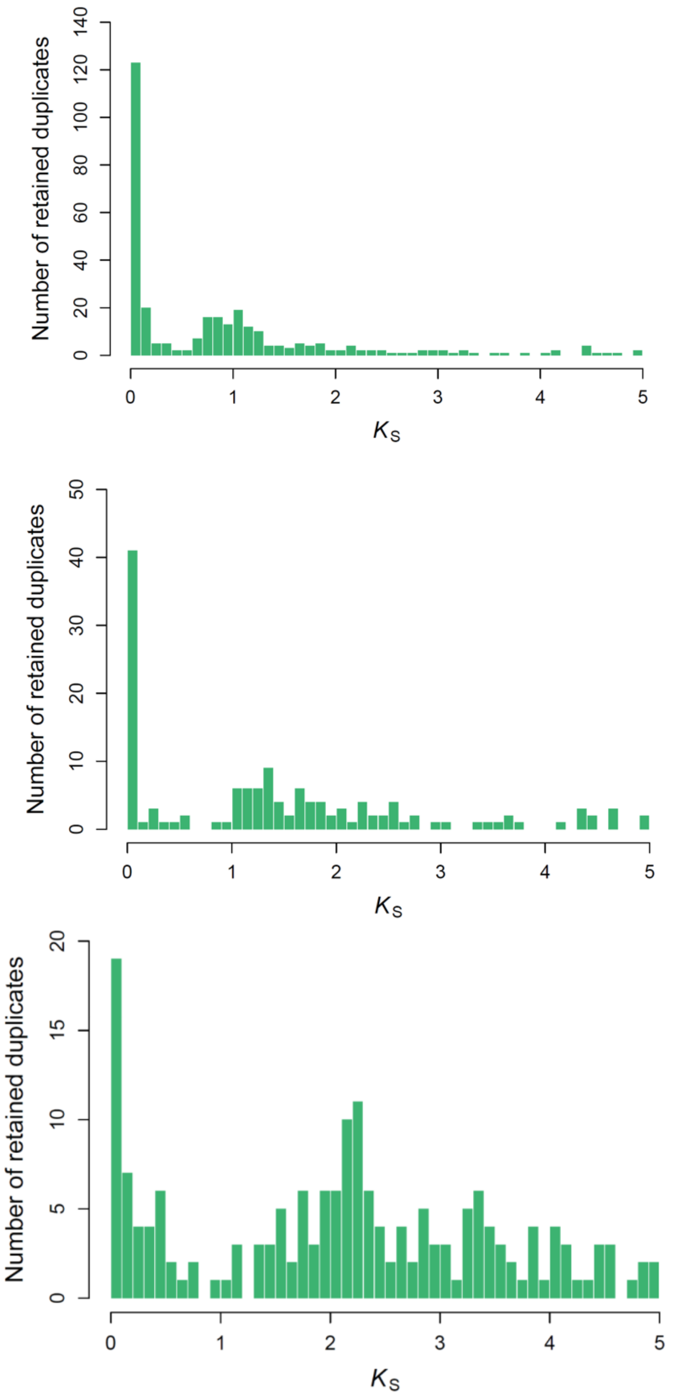

Fig. S8 K_S distributions for anchor pairs identified in *Azolla filiculoides* (upper), *Salvinia cucullata* (middle), and *Adiantum capillus-veneris* (lower). Anchor pairs with K_S values less than 0.1 tend to be located on short scaffolds in the genome assemblies of *Azolla filiculoides* and *Salvinia cucullata* (see Materials and Methods and Fig. S9).

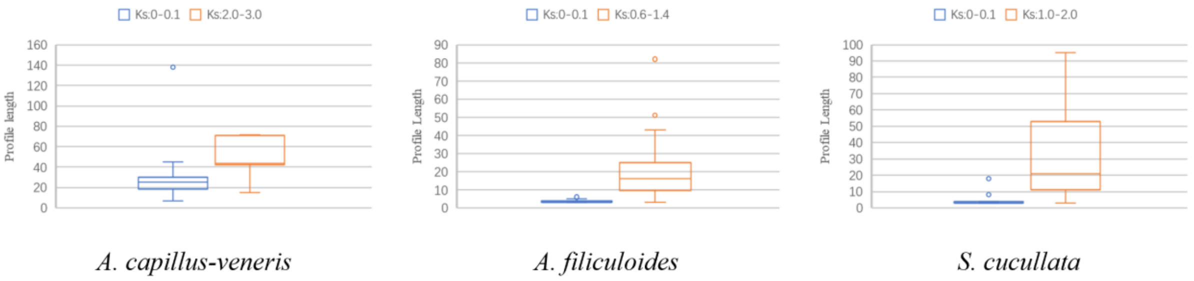

Fig. S9 Box plots of the number of genes without tandem duplicates (profile lengths) on scaffolds having anchor pairs with K_S values less than 0.1 and those having anchor pairs with K_S values near a potential WGD peak in the three fern genomes. The line in the middle of a box represents the median value and the top and bottom borders of the boxes denote the 75th and 25th percentiles, respectively. The upper and lower bars show the largest value within 1.5 times the interquartile range above the 75th percentile and the smallest value within 1.5 times the interquartile range below the 25th percentile, respectively. A dot shows the outside value, which is >1.5 times and <3 times the interquartile range beyond either end of the box.

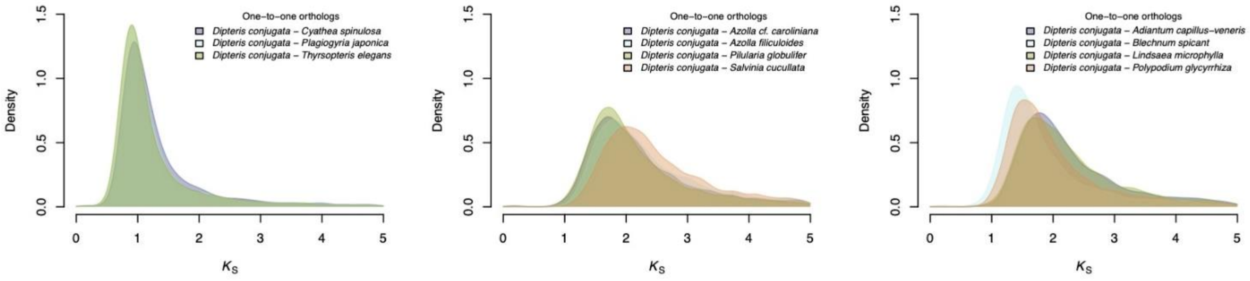

Fig. S10 The one-to-one orthologous K_S age distributions between *Dipteris conjugata* and species from Cyatheales (left), Salviniales (middle), and Polypodiales (right).

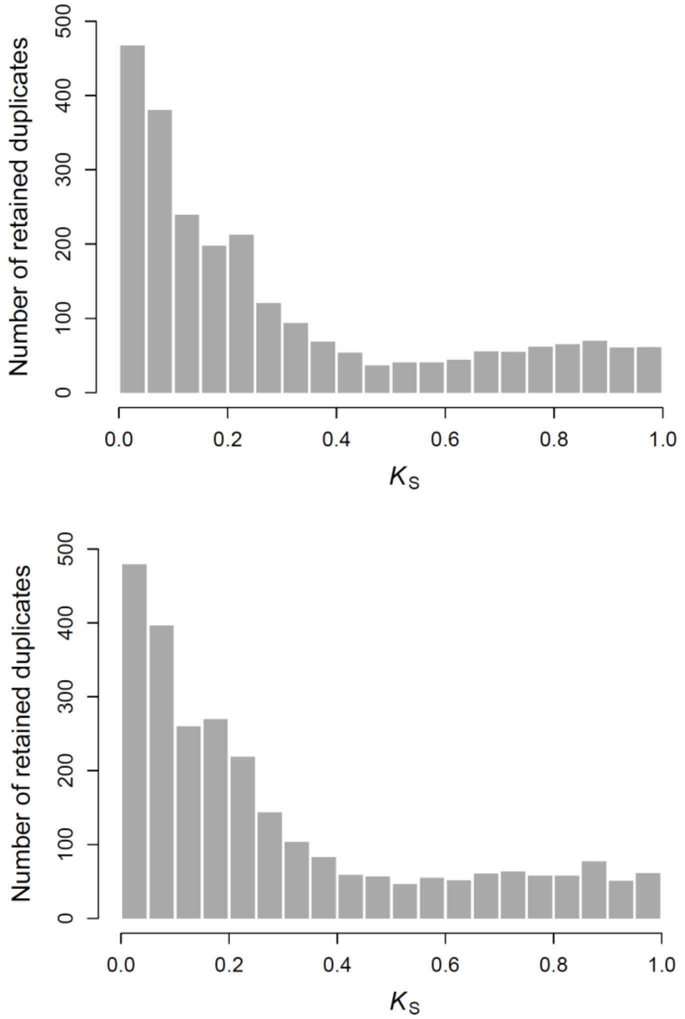

Fig. S11 The K_S distribution for paranomes of *Thyrsopteris elegans* (upper) and *Plagiogyria japonica* (lower) within a K_S range of [0,1.0] and a binwidth of 0.05.

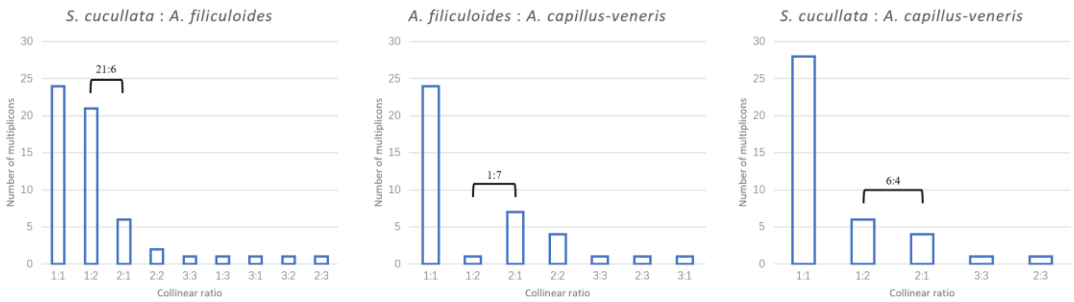

Fig. S12 Ratios of collinear blocks for pairwise intergenomic comparisons among the three genome-available ferns (see Materials and Methods). Collinear blocks with at least two species were retrieved and counted for the ratios, which turn out to be 2 : 1 : 1 for *Azolla filiculoides* : *Salvinia cucullata* : *Adiantum capillus-veneris*. The number above bars show the number of collinear blocks for a certain collinear ratio.

Table S1 Taxonomy, number of genes/unigenes and data source of fern species involved in this study.

Clade	Order	Family	Species	Number of genes/unigenes	Source of data
**Core Leptosporangiates**	Cyatheales	Cyatheaceae	*Cyathea spinulosa*	15288	1KP initiative (2019)
**Core Leptosporangiates**	Cyatheales	Thyrsopteridaceae	*Thyrsopteris elegans*	20444	1KP initiative (2019)
**Leptosporangiates**	Gleicheniales	Gleicheniaceae	*Sticherus lobatus*	2789	1KP initiative (2019)
**Leptosporangiates**	Gleicheniales	Dipteridaceae	*Dipteris conjugata*	18791	1KP initiative (2019)
**Leptosporangiates**	Osmundales	Osmundaceae	*Osmunda javanica*	14952	1KP initiative (2019)
**Core Leptosporangiates**	Cyatheales	Plagiogyriaceae	*Plagiogyria japonica*	21769	1KP initiative (2019)
**Core Leptosporangiates**	Polypodiales	Polypodiaceae	*Polypodium glycyrrhiza*	18785	1KP initiative (2019)
**Core Leptosporangiates**	Polypodiales	Blechnaceae	*Blechnum spicant*	18308	1KP initiative (2019)
**Core Leptosporangiates**	Polypodiales	Lindsaeaceae	*Lindsaea microphylla*	17074	1KP initiative (2019)
**Core Leptosporangiates**	Salviniales	Salviniaceae	*Azolla cf. caroliniana*	21852	1KP initiative (2019)
**Core Leptosporangiates**	Salviniales	Marsileaceae	*Pilularia globulifera*	18655	1KP initiative (2019)
**Leptosporangiates**	Schizaeales	Anemiaceae	*Anemia tomentosa*	19274	1KP initiative (2019)
**Leptosporangiates**	Schizaeales	Lygodiaceae	*Lygodium japonicum*	13716	1KP initiative (2019)
**Core Leptosporangiates**	Cyatheales	Cyatheaceae	*Cyathea spinulosa*	56985	Huang et al. (2020)
**Core Leptosporangiates**	Cyatheales	Thyrsopteridaceae	*Thyrsopteris elegans*	39658	Huang et al. (2020)

**Leptosporangiates**	Gleicheniales	Gleicheniaceae	*Sticherus truncatus*	35205	Huang et al. (2020)
**Leptosporangiates**	Gleicheniales	Dipteridaceae	*Dipteris conjugata*	26737	Huang et al. (2020)
**Leptosporangiates**	Osmundales	Osmundaceae	*Plenasium banksiaefolium*	26583	Huang et al. (2020)
**Core Leptosporangiates**	Cyatheales	Plagiogyriaceae	*Plagiogyria assurgens*	21829	Huang et al. (2020)
**Core Leptosporangiates**	Polypodiales	Polypodiaceae	*Polypodium virginianum*	28943	Huang et al. (2020)
**Core Leptosporangiates**	Polypodiales	Blechnaceae	*Blechnopsis orientalis*	30101	Huang et al. (2020)
**Core Leptosporangiates**	Polypodiales	Lindsaeaceae	*Lindsaea heterophylla*	34154	Huang et al. (2020)
**Core Leptosporangiates**	Salviniales	Salviniaceae	*Salvinia natans*	45028	Huang et al. (2020)
**Core Leptosporangiates**	Salviniales	Salviniaceae	*Azolla pinnata*	31501	Huang et al. (2020)
**Core Leptosporangiates**	Salviniales	Marsileaceae	*Marsilea quadrifolia*	26751	Huang et al. (2020)
**Leptosporangiates**	Schizaeales	Anemiaceae	*Anemia phyllitidis*	30786	Huang et al. (2020)
**Leptosporangiates**	Schizaeales	Lygodiaceae	*Lygodium japonicum*	21975	Huang et al. (2020)
**Core Leptosporangiates**	Salviniales	Salviniaceae	*Salvinia cucullata*	19780	Li et al. (2018)
**Core Leptosporangiates**	Salviniales	Salviniaceae	*Azolla filiculoides*	20203	Li et al. (2018)
**Core Leptosporangiates**	Polypodiales	Pteridaceae	*Adiantum capillus-veneris*	31244	

Table S2 The mean, standard deviation (SD), Monte Carlo standard error (MCSE), effective sample size (ESS), and 95% uncertainty interval for parameters estimated under the critical branch-specific DL+WGD model.

Parameters	mean	SD	MCSE	ESS	95% Uncertainty Interval
q1	0.029	0.006	0.000	530.377	0.018, 0.040
q2	0.058	0.005	0.000	574.912	0.048, 0.069
q3	0.263	0.008	0.000	482.108	0.247, 0.276
q4	0.193	0.011	0.000	610.220	0.170, 0.213
q5	0.006	0.004	0.000	468.739	0.000, 0.016
q6	0.000	0.000	0.000	615.278	0.000, 0.001
q7	0.142	0.008	0.001	438.831	0.126, 0.158
q8	0.027	0.008	0.000	268.830	0.010, 0.042
λ[1]	-2.173	0.031	0.002	701.823	-2.242, -2.114
λ[2]	-1.408	0.120	0.004	539.005	-1.658, -1.174
λ[3]	-2.430	0.040	0.002	323.043	-2.507, -2.355
λ[4]	-1.973	0.035	0.001	276.479	-2.049, -1.911
λ[5]	-2.377	0.046	0.002	433.675	-2.466, -2.290
λ[6]	-1.808	0.024	0.001	564.739	-1.849, -1.765
λ[7]	-2.351	0.055	0.002	386.449	-2.456, -2.238
λ[8]	-1.208	0.024	0.001	511.750	-1.257, -1.162
λ[9]	-1.317	0.029	0.002	285.137	-1.375, -1.264
λ[10]	-1.668	0.034	0.002	421.787	-1.735, -1.601
λ[11]	-1.977	0.030	0.001	655.696	-2.033, -1.921
λ[12]	-1.831	0.036	0.002	691.116	-1.901, -1.764
λ[13]	-1.318	0.041	0.003	507.199	-1.404, -1.235
λ[14]	-0.551	0.026	0.001	582.657	-0.599, -0.500
λ[15]	-2.425	0.045	0.001	924.685	-2.507, -2.347
λ[16]	-1.758	0.027	0.001	625.221	-1.817, -1.704
λ[17]	-3.574	0.364	0.026	230.203	-4.434, -2.964
λ[18]	-1.891	0.062	0.003	474.721	-2.012, -1.774
λ[19]	-1.612	0.066	0.002	546.528	-1.730, -1.486
λ[20]	-0.855	0.018	0.001	579.013	-0.890, -0.818
λ[21]	-5.313	0.517	0.025	469.554	-6.407, -4.390
λ[22]	-1.158	0.022	0.001	533.635	-1.198, -1.114
λ[23]	-1.908	1.012	0.026	507.651	-4.023, 0.182
λ[24]	-1.606	0.086	0.004	550.205	-1.772, -1.444
λ[25]	-1.730	0.034	0.001	518.691	-1.798, -1.666
λ[26]	-0.971	0.020	0.000	617.539	-1.008, -0.934
λ[27]	0.753	0.029	0.002	487.080	0.700, 0.810
λ[28]	-1.710	0.106	0.004	228.761	-1.933, -1.510
λ[29]	-0.627	0.040	0.002	725.915	-0.713, -0.545

λ[30]	-4.076	0.569	0.017	264.698	-5.457, -3.175
σ	0.996	0.133	0.007	299.282	0.779, 1.276
r	-1.830	0.194	0.008	594.378	-2.203, -1.452
η	0.728	0.005	0.000	510.286	0.718, 0.738

Table S3 The mean, standard deviation (SD), Monte Carlo standard error (MCSE), effective sample size (ESS), and 95% uncertainty interval for parameters estimated under the relaxed branch-specific DL+WGD model.

Parameters	mean	SD	MCSE	ESS	95% Uncertainty Interval
q1	0.001	0.001	0.000	415.591	0.000, 0.002
q2	0.028	0.007	0.001	752.973	0.017, 0.043
q3	0.352	0.014	0.000	777.914	0.328, 0.380
q4	0.170	0.022	0.000	424.251	0.127, 0.206
q5	0.156	0.014	0.000	465.288	0.131, 0.182
q6	0.020	0.010	0.001	681.184	0.001, 0.038
q7	0.127	0.010	0.001	220.394	0.106, 0.145
q8	0.025	0.010	0.000	515.911	0.001, 0.039
λ[1]	-2.131	0.033	0.002	386.117	-2.184, -2.063
μ[1]	-0.930	0.026	0.002	313.478	-0.979, -0.883
λ[2]	-1.038	0.042	0.002	749.225	-1.113, -0.962
μ[2]	-5.706	0.557	0.021	556.223	-6.890, -4.850
λ[3]	-2.275	0.032	0.003	622.471	-2.338, -2.213
μ[3]	-2.978	0.122	0.010	504.486	-3.291, -2.805
λ[4]	-1.787	0.029	0.003	326.513	-1.844, -1.734
μ[4]	-7.129	0.625	0.025	346.896	-8.271, -5.851
λ[5]	-1.647	0.030	0.003	334.439	-1.707, -1.587
μ[5]	-8.243	0.730	0.025	583.223	-9.416, -7.012
λ[6]	-1.755	0.025	0.002	231.712	-1.805, -1.708
μ[6]	-2.280	0.051	0.003	528.412	-2.372, -2.172
λ[7]	-2.263	0.103	0.009	250.742	-2.455, -2.044
μ[7]	-2.657	0.081	0.009	296.198	-2.792, -2.512
λ[8]	-1.150	0.025	0.001	213.373	-1.192, -1.099
μ[8]	-2.380	0.130	0.010	665.241	-2.649, -2.122
λ[9]	-1.212	0.031	0.003	555.807	-1.277, -1.153
μ[9]	-3.497	0.359	0.015	270.643	-4.421, -2.774
λ[10]	-1.566	0.036	0.004	357.115	-1.638, -1.497
μ[10]	-4.042	0.666	0.019	335.758	-5.603, -3.020
λ[11]	-1.860	0.027	0.003	243.793	-1.913, -1.816
μ[11]	-2.952	0.121	0.009	799.713	-3.258, -2.776
λ[12]	-1.751	0.031	0.003	782.521	-1.804, -1.692
μ[12]	-2.948	0.153	0.008	302.534	-3.314, -2.720
λ[13]	-1.525	0.038	0.004	794.917	-1.591, -1.455
μ[13]	-1.722	0.098	0.009	569.252	-1.959, -1.521
λ[14]	-0.057	0.026	0.002	309.481	-0.103, -0.010
μ[14]	-7.587	0.660	0.022	390.974	-8.843, -6.391
λ[15]	-1.908	0.034	0.003	858.727	-1.979, -1.845

μ[15]	-8.360	0.519	0.016	454.628	-9.300, -7.395
λ[16]	-1.547	0.021	0.002	804.907	-1.588, -1.511
μ[16]	-7.844	0.655	0.020	453.347	-9.015, -6.543
λ[17]	-4.157	0.094	0.004	689.199	-4.381, -3.871
μ[17]	-5.266	0.641	0.022	342.244	-6.817, -4.126
λ[18]	-3.236	0.125	0.008	815.800	-3.466, -2.991
μ[18]	-1.423	0.054	0.005	404.954	-1.518, -1.308
λ[19]	-2.990	0.139	0.010	213.475	-3.307, -2.757
μ[19]	-0.976	0.055	0.006	334.112	-1.078, -0.874
λ[20]	-2.430	0.064	0.003	474.989	-2.554, -2.316
μ[20]	-0.195	0.014	0.001	742.153	-0.221, -0.168
λ[21]	-6.857	0.584	0.021	399.682	-8.266, -5.960
μ[21]	-2.406	0.022	0.001	521.978	-2.437, -2.359
λ[22]	-3.053	0.034	0.001	452.717	-3.121, -3.016
μ[22]	-0.596	0.016	0.002	420.542	-0.625, -0.569
λ[23]	-2.741	0.059	0.005	563.534	-2.858, 2.632
μ[23]	-2.413	0.036	0.002	619.645	-2.477, 2.370
λ[24]	-2.114	0.151	0.010	633.701	-2.348, -1.835
μ[24]	-1.890	0.097	0.009	414.970	-2.098, -1.696
λ[25]	-3.733	0.187	0.004	708.231	-4.069, -3.350
μ[25]	-1.473	0.035	0.004	825.114	-1.540, -1.408
λ[26]	-3.663	0.120	0.003	519.978	-3.919, -3.457
μ[26]	-0.202	0.015	0.001	752.811	-0.233, -0.172
λ[27]	1.054	0.033	0.004	638.188	0.994, 1.112
μ[27]	0.324	0.081	0.009	305.529	0.176, 0.471
λ[28]	-4.566	0.113	0.005	752.789	-4.758, -4.387
μ[28]	-0.473	0.032	0.003	806.923	-0.534, -0.415
λ[29]	-0.444	0.058	0.007	860.680	-0.558, -0.350
μ[29]	-0.832	0.079	0.008	492.495	-0.989, -0.701
λ[30]	-6.581	0.631	0.022	524.719	-8.026, -5.589
μ[30]	-1.147	0.280	0.009	412.916	-1.717, -0.702
ρ	-0.130	0.218	0.009	351.196	-0.567, 0.325
τ	2.399	0.174	0.008	311.640	2.117, 2.759
η	0.738	0.005	0.001	765.294	0.730, 0.747

Table S4 The mean, standard deviation (SD), Monte Carlo standard error (MCSE), effective sample size (ESS), and 95% uncertainty interval for parameters estimated under the critical branch-specific DL+WGD model for the randomly selected gene families.

Parameters	mean	SD	MCSE	ESS	95% Uncertainty Interval
q1	0.0899	0.0223	0.0007	456.1758	0.046, 0.135
q2	0.0587	0.0167	0.0003	373.7697	0.023, 0.091
q3	0.2543	0.0189	0.0012	459.5596	0.218, 0.292
q4	0.2378	0.0297	0.0009	485.3025	0.181, 0.299
q5	0.0551	0.0263	0.0014	300.3996	0.009, 0.110
q6	0.0018	0.0017	0.0001	405.0572	0.000, 0.006
q7	0.1421	0.0235	0.0009	636.6395	0.095, 0.185
q8	0.0496	0.0196	0.0007	358.1861	0.013, 0.089
λ[1]	-2.3617	0.0811	0.0026	867.9382	-2.522, -2.199
λ[2]	-1.1958	0.2841	0.01	622.9037	-1.758, -0.685
λ[3]	-2.6393	0.105	0.0043	630.1546	-2.856, -2.441
λ[4]	-1.8881	0.0846	0.0029	635.0129	-2.052, -1.721
λ[5]	-2.3223	0.1166	0.006	460.253	-2.549, -2.095
λ[6]	-1.8816	0.0655	0.0023	638.5018	-2.008, -1.759
λ[7]	-2.7303	0.1925	0.0062	689.3348	-3.094, -2.393
λ[8]	-1.1936	0.0704	0.002	669.8621	-1.332, -1.058
λ[9]	-1.3414	0.0783	0.0031	692.1576	-1.516, -1.193
λ[10]	-1.9383	0.1167	0.0031	906.8149	-2.180, -1.719
λ[11]	-2.0515	0.0756	0.0029	630.8564	-2.217, -1.921
λ[12]	-1.6796	0.0865	0.0036	712.5232	-1.854, -1.511
λ[13]	-1.2469	0.0967	0.0043	715.8083	-1.438, -1.066
λ[14]	-0.4991	0.0717	0.0025	734.7804	-0.643, -0.365
λ[15]	-2.4859	0.1202	0.0044	785.3068	-2.742, -2.256
λ[16]	-1.9077	0.0766	0.0035	681.7638	-2.055, -1.762
λ[17]	-3.2501	0.4933	0.0249	556.8627	-4.363, -2.430
λ[18]	-2.3346	0.2262	0.0085	466.5147	-2.824, -1.942
λ[19]	-1.664	0.1802	0.0057	688.6849	-2.059, -1.330
λ[20]	-1.0717	0.059	0.0026	511.648	-1.180, -0.957
λ[21]	-3.5624	0.5525	0.0233	398.3133	-4.879, -2.701
λ[22]	-1.394	0.0796	0.0031	639.1386	-1.546, -1.246
λ[23]	-1.8863	0.8076	0.0321	517.775	-3.620, -0.348
λ[24]	-1.9174	0.2872	0.012	486.5428	-2.495, -1.386
λ[25]	-1.797	0.1054	0.0038	589.2052	-1.999, -1.594
λ[26]	-1.2224	0.0712	0.0024	701.3025	-1.367, -1.093
λ[27]	0.3118	0.112	0.0048	496.124	0.080, 0.542
λ[28]	-2.5745	0.4745	0.022	491.7906	-3.700, -1.816

λ[29]	-0.9442	0.1447	0.0064	440.5323	-1.257, -0.688
λ[30]	-3.3458	0.5312	0.0214	472.0908	-4.489, -2.433
σ	0.8032	0.1101	0.0044	457.4962	0.617, 1.058
r	-1.8717	0.1519	0.0061	469.3254	-2.191, -1.592
η	0.8182	0.0149	0.0005	574.1978	0.789, 0.848

Table S5 The mean, standard deviation (SD), Monte Carlo standard error (MCSE), effective sample size (ESS), and 95% uncertainty interval for parameters estimated under the relaxed branch-specific DL+WGD model for the randomly selected gene families.

Parameters	mean	SD	MCSE	ESS	95% Uncertainty Interval
q1	0.0043	0.004	0.0002	453.7002	0.000, 0.015
q2	0.0458	0.0233	0.0014	267.187	0.006, 0.095
q3	0.349	0.0304	0.0021	205.7778	0.293, 0.408
q4	0.1533	0.0591	0.0042	225.8308	0.033, 0.253
q5	0.1261	0.0362	0.0017	347.524	0.045, 0.191
q6	0.0302	0.0142	0.0009	249.0382	0.002, 0.055
q7	0.1247	0.0266	0.0014	278.8533	0.070, 0.173
q8	0.0174	0.0105	0.0006	336.6516	0.001, 0.040
λ[1]	-2.1733	0.0884	0.0059	242.2756	-2.340, -2.005
μ[1]	-1.183	0.1159	0.0074	329.038	-1.417, -0.973
λ[2]	-0.8451	0.0965	0.0072	266.0184	-1.033, -0.638
μ[2]	-4.6579	1.4606	0.1101	183.8768	-7.954, -2.340
λ[3]	-2.3576	0.0921	0.0046	461.8257	-2.543, -2.190
μ[3]	-5.147	0.8717	0.0508	294.9318	-7.082, -3.824
λ[4]	-1.6586	0.0777	0.0035	484.7828	-1.806, -1.505
μ[4]	-5.7084	1.057	0.0526	352.8704	-8.191, -4.036
λ[5]	-1.6539	0.0963	0.0038	555.8048	-1.855, -1.483
μ[5]	-6.4705	1.0031	0.0496	298.349	-8.529, -4.788
λ[6]	-1.7485	0.0639	0.0028	559.4425	-1.876, -1.631
μ[6]	-2.7406	0.1822	0.0092	421.6705	-3.148, -2.444
λ[7]	-2.3685	0.2844	0.0176	266.0791	-2.904, -1.837
μ[7]	-4.2019	0.9977	0.0711	156.0238	-6.685, -2.866
λ[8]	-1.1603	0.0682	0.003	439.2458	-1.298, -1.038
μ[8]	-2.7667	0.6123	0.0367	207.8692	-4.612, -2.054
λ[9]	-1.1868	0.0651	0.003	417.8248	-1.323, -1.068
μ[9]	-4.9963	1.2217	0.0687	253.0961	-7.708, -3.087
λ[10]	-1.5785	0.0885	0.0045	408.7491	-1.759, -1.414
μ[10]	-5.5596	1.1002	0.0566	392.4962	-7.997, -3.834
λ[11]	-1.9051	0.0702	0.002	748.8113	-2.049, -1.775
μ[11]	-4.4912	0.9229	0.0621	145.2772	-6.659, -3.194
λ[12]	-1.5212	0.0879	0.0041	530.176	-1.689, -1.357
μ[12]	-3.7849	0.9608	0.0672	193.4022	-6.190, -2.543
λ[13]	-1.3598	0.1099	0.0051	527.2714	-1.595, -1.142
μ[13]	-2.2912	0.5487	0.0446	151.6815	-3.477, -1.562
λ[14]	-0.0344	0.055	0.0032	405.1462	-0.135, 0.069

μ[14]	-5.6978	1.3458	0.0769	264.1519	-8.700, -3.494
λ[15]	-1.912	0.0864	0.0036	684.9452	-2.081, -1.747
μ[15]	-6.4972	1.0335	0.0481	387.9386	-8.685, -4.773
λ[16]	-1.5931	0.0623	0.0037	357.739	-1.713, -1.470
μ[16]	-6.3422	1.0175	0.0615	263.69	-8.704, -4.747
λ[17]	-4.3576	1.0183	0.062	251.3392	-6.753, -2.685
μ[17]	-4.2135	1.2002	0.0895	151.4352	-7.282, -2.595
λ[18]	-3.9801	0.5872	0.0379	236.6547	-5.184, -3.001
μ[18]	-1.7907	0.1584	0.0071	492.7281	-2.117, -1.512
λ[19]	-2.8813	0.3955	0.0164	331.9864	-3.744, -2.199
μ[19]	-1.0248	0.1494	0.007	478.983	-1.327, -0.761
λ[20]	-2.9354	0.2128	0.0116	399.526	-3.372, -2.580
μ[20]	-0.3479	0.0476	0.0028	450.819	-0.435, -0.245
λ[21]	-5.0817	1.1164	0.0668	258.96	-7.691, -3.379
μ[21]	-2.3623	0.7346	0.0582	140.1146	-4.239, -1.414
λ[22]	-3.241	0.632	0.0343	330.9183	-4.644, -2.250
μ[22]	-0.7547	0.0552	0.0034	307.2742	-0.867, -0.651
λ[23]	-2.0139	2.1166	0.0947	515.6217	-5.962, 2.662
μ[23]	-3.1112	2.0755	0.1113	389.3996	-6.885, 0.888
λ[24]	-3.0385	0.8845	0.0448	333.8142	-5.372, -1.665
μ[24]	-2.2244	0.4065	0.0246	266.4421	-3.163, -1.591
λ[25]	-4.2875	1.1146	0.0691	352.7197	-7.382, -2.903
μ[25]	-1.5869	0.0944	0.0042	456.6939	-1.774, -1.389
λ[26]	-4.7919	0.7041	0.0371	383.4005	-6.327, -3.707
μ[26]	-0.3425	0.0474	0.003	342.4763	-0.435, -0.256
λ[27]	0.7156	0.1169	0.0086	196.9298	0.490, 0.941
μ[27]	-0.7525	0.4628	0.0381	157.5094	-1.770, -0.099
λ[28]	-4.1086	1.083	0.0658	334.0481	-6.510, -2.522
μ[28]	-0.8821	0.1219	0.0058	536.7204	-1.116, -0.640
λ[29]	-0.8449	0.2509	0.0156	258.6572	-1.390, -0.446
μ[29]	-1.288	0.1789	0.0079	453.0615	-1.657, -0.972
λ[30]	-4.4117	1.1245	0.071	259.0136	-6.883, -2.534
μ[30]	-5.3595	1.0669	0.0461	527.4255	-7.938, -3.701
ρ	-0.2171	0.1898	0.012	298.7117	-0.559, 0.203
τ	1.9711	0.249	0.0214	129.0709	1.576, 2.590
η	0.8077	0.0107	0.0005	350.4232	0.787, 0.829

Methods S1 Julia code for the Whale analyses with the critical and relaxed branch-specific DL+WGD models.

```
$## Basic model setting and data reading
$using Pkg; Pkg.activate(@__DIR__)
$using Whale, NewickTree, Turing, DataFrames, CSV, JSON, $Serialization,
LinearAlgebra, Optim, Distributions
$tree = readnw(readline("species_list.nwk"))
$for n in postwalk(tree)
$    n.data.distance /= 100
$end
$nn = length(postwalk(tree))
$insertnode!(getlca(tree, "Blechnum-spicant", "Pilularia-$globulifera"),
name="wgd_1")
$insertnode!(getlca(tree, "Cyathea-spinulosa","Plagiogyria-$japonica"),
name="wgd_2")
$insertnode!(getlca(tree, "Blechnum-spicant", "Lygodium-$japonicum"),
name="wgd_3")
$insertnode!(getlca(tree, "Lygodium-japonicum","Anemia-$tomentosa"),
name="wgd_4")
$insertnode!(getlca(tree, "Lindsaea-microphylla"), name="wgd_5")
$insertnode!(getlca(tree, "Azolla-filiculoides","Azolla-cf-$caroliniana"),
name="wgd_6")
$insertnode!(getlca(tree, "Azolla-filiculoides","Salvinia-$cucullata"),
name="wgd_7")
$insertnode!(getlca(tree, "Azolla-filiculoides","Dipteris-$conjugata"),
name="wgd_8")
$param = ConstantDLWGD(λ=1., μ=1., η=0.9, q=[0.1, 0.1, 0.1, 0.1,
$0.1,0.1,0.1,0.1],p=[0.202,0.940,0.292,0.222,0.280,0.118,0.399,0$.366,0.426,0
.553,0.286,0.228,0.201,0.426,0.499,0.409])
$model = WhaleModel(param, tree, .1, $condition=Whale.RootCondition(),
minn=5, maxn=10)
$data = read_ale("nf-out", model)
$out = mkpath("output")
$write(joinpath(out, "model.txt"), repr(model))


$## The model setting of critical branch-specific DL+WGD model
$@model critical(model, X) = begin
$    η ~ truncated(Beta(3,1), 0.01, 0.99)
$    r ~ Turing.Flat()
$    σ ~ Exponential(0.1)
$    λ ~ MvNormal(repeat([r], nn-1), σ)
$    l = [λ; r]
$    q1 ~ Beta()
$    q2 ~ Beta()
$    q3 ~ Beta()
$    q4 ~ Beta()
```

```
$    q5 ~ Beta()
$    q6 ~ Beta()
$    q7 ~ Beta()
$    q8 ~ Beta()
$    X ~ model((λ=1, μ=1, η=η, q=[q1,q2,q3,q4,q5,q6,q7,q8]))
$end
$rates = DLWGD(λ=zeros(nn), μ=zeros(nn), η=0.9,
$q=[0.1,0.1,0.1,0.1,0.1,0.1,0.1,0.1],p=[0.202,0.940,0.292,0.222,
$0.280,0.118,0.399,0.366,0.426,0.553,0.286,0.228,0.201,0.426,0.4$99,0.409])
$model = WhaleModel(rates, tree, .1, minn=5, maxn=10,
$condition=Whale.RootCondition())
$chaincritical = sample(critical(model, data), NUTS(0.65), 500)
$CSV.write(joinpath(out, "chaincritical.csv"), chaincritical)
$serialize(joinpath(out, "chaincritical.jls"), chaincritical)

$## The model setting of relaxed branch-specific DL+WGD model
$@model branchrates(model, X, n, τmean=1.) = begin
$    η ~ Beta(3,1)
$    ρ ~ Uniform(-1, 1.)
$    τ ~ Exponential(τmean)
$    T = typeof(ρ)
$    S = [τ 0. ; 0. τ]
$    R = [1. ρ ; ρ 1.]
$    Σ = S*R*S
$    !isposdef(Σ) && return -Inf
$    r = Matrix{T}(undef, 2, n)
$    o = id(getroot(model))
$    r[:,o] ~ MvNormal(zeros(2), ones(2))
$    for i=1:n
$        i == o && continue
$        r[:,i] ~ MvNormal(r[:,o], Σ)
$    end
$    q1 ~ Beta()
$    q2 ~ Beta()
$    q3 ~ Beta()
$    q4 ~ Beta()
$    q5 ~ Beta()
$    q6 ~ Beta()
$    q7 ~ Beta()
$    q8 ~ Beta()
$    X ~ model((λ=r[1,:], μ=r[2,:], η=η,
$q=[q1,q2,q3,q4,q5,q6,q7,q8],p=[0.202,0.940,0.292,0.222,0.280,0.
$118,0.399,0.366,0.426,0.553,0.286,0.228,0.201,0.426,0.499,0.409$]))
$end
$bmodel = branchrates(model, data, nn)
$chainrelaxed = sample(bmodel, NUTS(0.65), 500)
$CSV.write(joinpath(out, "chainrelaxed.csv"), chainrelaxed)
$serialize(joinpath(out, "chainrelaxed.jls"), chainrelaxed)
$##End
```

Methods S2 Julia code for the Whale analysis of gene tree – species tree reconciliations.

```
$## Basic model setting and data reading
$using Pkg; Pkg.activate(@__DIR__)
$using Whale, NewickTree, Turing, DataFrames, CSV, JSON, $Serialization,
LinearAlgebra, Optim, Distributions
$tree2  = readnw(readline("species_list.nwk"))
$for n in postwalk(tree2)[1:end-1]
$    n.data.distance = 1.
$end
$ccd = read_ale("nf-out", model)
```

```
$## The model setting of mere reconciliation DL model
$@model distance(model, ccd, n, η=0.75) = begin
$    r0 ~ Turing.FlatPos(0.)
$    T = typeof(r0)
$    r = Vector{T}(undef, n)
$    o = getroot(model)
$    r[id(o)] = r0
$    for n in model.order
$        isroot(n) && continue
$        r[id(n)] ~ Exponential(r0)
$    end
$    ccd ~ model((λ=log.(r), μ=log.(r), q=T[], η=η))
$end
$reconciliation = sample(distance(model, ccd, n), NUTS(),500)
$CSV.write(joinpath(out, "reconciliation.csv"), reconciliation)
$serialize(joinpath(out, "reconciliation.jls"), reconciliation)
$##End
```
